# Polypharmacy and quality of life in Yemeni cancer patients undergoing chemotherapy: A cross-sectional study

**DOI:** 10.1371/journal.pgph.0006281

**Published:** 2026-09-28

**Authors:** Sayida Al-Jamei, Haneen Al-ansi, Malak Albiel, Enas Al-Awadhi, Shaima Jihad, Amal Noman, Fadwa Alhayani, Rehab Alarhabi

**Affiliations:** Department of Clinical Pharmacy, School of Medical Science and Pharmacy, Lebanese International University, Sana’a, Yemen; Wits University: University of the Witwatersrand Johannesburg, SOUTH AFRICA

## Abstract

Polypharmacy is common among cancer patients receiving chemotherapy due to the need for multiple supportive medications. This study assessed the prevalence of non-oncological polypharmacy and its association with quality of life (QoL) among adult cancer patients in Yemen. A cross-sectional study was conducted between 1st December 2025 and 23rd February 2026 at the National Oncology Centre in Sana’a, Yemen. Adult patients (≥18 years) receiving chemotherapy were recruited using convenience sampling. Data were collected through face-to-face interviews and medical record review. Polypharmacy was defined as the use of ≥5 non-oncological medications and excessive polypharmacy as ≥10 medications. QoL was assessed using the Arabic version of the EORTC QLQ-C30. Statistical analyses included descriptive statistics, independent-sample *t*-tests, and multiple linear regression using IBM SPSS Statistics version 27. Among 234 participants, 63.2% were female and 47.6% were aged 31–50 years. Polypharmacy was highly prevalent: 94.9% used ≥5 non-oncological medications and 56.4% ≥ 10. Mean global QoL was 47.9 (SD 26.7). Patients reported substantial financial difficulties (mean 82.3), fatigue (61.1), and pain (59.6). QoL did not differ significantly between excessive polypharmacy groups (mean difference −3.35; 95% CI −10.58 to 3.87; p = 0.361). In multivariable analyses, female gender independently predicted lower QoL (B = −10.13; 95% CI: −18.19 to −2.07; p = 0.014) and higher non-oncological medication use (B = 1.18; 95% CI: 0.25 to 2.10; p = 0.013). Age ≥ 51 years and illiteracy showed borderline associations with lower QoL, while all other variables, including excessive polypharmacy (≥10 non-oncological medications), were not significantly associated with QoL. Non-oncological Polypharmacy is highly prevalent among Yemeni cancer patients receiving chemotherapy. Although medication burden was substantial, it was not independently associated with QoL, whereas female gender predicted poorer outcomes.

## 1. Introduction

Cancer is currently the second leading cause of premature death worldwide, following cardiovascular diseases. According to World Health Organization estimates, cancer is projected to surpass cardiovascular disease as the primary cause of premature death in many countries during this century, underscoring its growing importance as a major global public health challenge [1,2]. This burden is reflected in the rising global incidence and mortality of cancer: in 2022, nearly 20 million new cases and approximately 9.7 million cancer-related deaths were reported, with projections for 2040 estimating 29.9 million new cases and 15.3 million deaths [3].

In Yemen, cancer represents a significant public health concern, although detailed epidemiological data remain limited due to scarce pathology resources, incomplete medical records, and a lack of comprehensive population-based studies. According to available data, the crude incidence rate of all cancers was 55.2 per 100,000 in 2020, corresponding to approximately 31,811 prevalent cases and 16,525 newly diagnosed cases nationally [2,4,5]. The ongoing conflict has further compounded this burden, leaving nearly half of healthcare facilities partially or completely inoperable, creating critical shortages of medical supplies, and limiting access to specialized treatments, including oncology services. Currently, only 149 of 5,042 healthcare facilities are fully functional, forcing many patients to travel long distances through hazardous conditions to access care [3].

Recent advances in cancer treatment—including immunotherapy, chemotherapy, molecularly targeted therapies, hormonal therapy, surgery, and radiotherapy—have significantly improved treatment outcomes and prolonged patient survival [6,7]. Among these modalities, chemotherapy remains a cornerstone of cancer treatment for many malignancies. However, chemotherapy is frequently associated with a wide range of adverse effects that may substantially affect patients’ well-being and daily functioning [8]. Previous studies have identified nausea, vomiting, fatigue, weakness, appetite loss, gastrointestinal symptoms, pain, and hematologic toxicity as some of the most common complications experienced by patients undergoing chemotherapy [9–11].

To manage these treatment‑related toxicities and coexisting medical conditions, cancer patients often require multiple medications simultaneously. The concurrent use of multiple medications, commonly referred to as polypharmacy, may increase the risk of adverse drug reactions and drug–drug interactions, particularly in oncology populations with complex therapeutic regimens and supportive care needs [12–14]. The World Health Organization defines polypharmacy as the concurrent use of multiple medications, including prescription drugs, over-the-counter medications, and Natural Products / Herbal and Traditional Remedies [15]. Although the most widely accepted definition involves the regular use of five or more medications, an even higher threshold—commonly referred to as excessive polypharmacy, defined as the use of 10 or more medications—has been increasingly recognized as a marker of heightened risk for adverse outcomes [16].

Polypharmacy is common among cancer patients of all ages [17–19]. In the general population, polypharmacy has been associated with a range of adverse health outcomes, including increased risk of adverse drug reactions, drug–drug interactions, cognitive decline, functional impairment, prolonged hospital stays, increased healthcare costs, and medication nonadherence [17,20,21]. In oncology settings, these risks may be amplified because cancer treatment often requires the addition of multiple supportive medications to manage treatment-related side effects and comorbid conditions [18,19].

Beyond its physical consequences, a cancer diagnosis can profoundly affect patients’ psychological and social well-being. Quality of life (QoL) frequently deteriorates following diagnosis due to increased anxiety, uncertainty about the future, and depressive symptoms, which are often influenced by societal perceptions of cancer as a life-threatening and debilitating disease [20,22,23]. Treatment-related toxicities may further compromise QoL across multiple domains, including physical, emotional, cognitive, and social functioning [24]. For example, perceived stigma, social withdrawal, and changes in interpersonal relationships may contribute to impaired social functioning, with these effects varying widely depending on individual psychological, social, and clinical factors [25].

Despite the growing recognition of polypharmacy as a major clinical concern in oncology care, relatively few studies have examined its relationship with quality of life among cancer patients. Existing research remains limited and is largely concentrated in high-income countries [26–28]. Evidence from low- and middle-income countries, particularly in the Middle East, remains scarce. To date, no published studies have specifically examined the prevalence of polypharmacy or its potential impact on quality of life among cancer patients in Yemen.

Therefore, the present study aimed to examine the prevalence and determinants of non-oncological polypharmacy among cancer patients undergoing chemotherapy in Yemen and to evaluate its association with health-related quality of life.

## 2. Methods

### 2.1. Ethics statement

This study was conducted in accordance with the ethical principles outlined in the Declaration of Helsinki and was approved by the Ethics Committee of Lebanese International University, Sana’a, Yemen (Approval No. LIU/UREC/2025/008). Written informed consent was obtained from all participants prior to enrollment. Given the vulnerability of cancer patients receiving chemotherapy, participation was strictly voluntary, and patients were informed that refusal would not affect their treatment or care. Interviews were conducted privately during routine visits and kept brief to reduce burden.

### 2.2. Study design, setting, and participants

This observational cross-sectional study was conducted between December 2025 and February 2026 at the National Oncology Center in Sana'a, Yemen, a principal referral institution for oncology care. Adult patients (≥18 years) with a confirmed cancer diagnosis who were actively receiving chemotherapy and had completed at least one chemotherapy cycle were eligible. Participants were recruited using convenience sampling from inpatient wards during routine treatment visits.

Patients were excluded if they were unaware of their cancer diagnosis, had incomplete medical records, were unable to complete face-to-face interviews because of cognitive or physical limitations, or declined participation.

Eligible patients were informed about the study objectives and procedures, and written informed consent was obtained prior to enrollment. Data were collected through structured face-to-face interviews, while clinical and medication-related information was verified from hospital medical records, including systemic anti-cancer therapies, supportive medications, and natural, herbal, and other complementary products used during treatment. Each interview lasted approximately 20–30 minutes.

### 2.3. Sample size calculation

Sample size adequacy was assessed using Green’s rule of thumb for multiple regression (N ≥ 50 + 8m), where m represents the number of predictors included in the regression model [29]. Based on this criterion, the minimum required sample size was 138 participants for the Global Health Status/Quality of Life (QoL) regression model, which included 11 predictors, and 130 participants for the medication burden regression model, which included 10 predictors. The final analytical sample of 234 participants exceeded these recommended thresholds.

### 2.4. Study instruments

Data were collected using a structured instrument to capture participants’ sociodemographic characteristics, clinical profile, medication use, and health-related quality of life. Sociodemographic and clinical data were obtained through face-to-face interviews and verified from medical records at the National Oncology Center. Participants were asked about age, gender, marital status, occupation, education, residence (urban/rural), and monthly income. Clinical data included cancer type, stage, time since diagnosis, comorbidities, and details of chemotherapy, including systemic anti-cancer therapy and number of cycles completed.

Medication data were recorded using a structured polypharmacy assessment form, including all current medications: systemic anti-cancer agents, supportive therapies (e.g., antiemetics, corticosteroids, analgesics, electrolytes, vitamins), chronic disease medications, over-the-counter products, and natural products/herbal and traditional remedies commonly used alongside cancer treatment (e.g., honey, garlic, ginger, black seed, and caper bush). The total number of medications per participant was calculated. However, systemic anti-cancer agents were excluded from the polypharmacy count to avoid overestimation of medication burden driven by protocol-based chemotherapy regimens that are not typically modifiable in routine clinical practice. This approach allows a more accurate assessment of concomitant (non-oncological) medication burden related to comorbid conditions and supportive care needs. Polypharmacy was defined as the concurrent use of five or more (≥5) non-oncological medications, with excessive polypharmacy defined as ten or more (≥10) non-oncological medications.

Quality of life was assessed using the validated Arabic version of the EORTC QLQ-C30 (Version 3.0), which includes 30 items covering five functional domains (physical, role, emotional, cognitive, social), three symptom scales (fatigue, pain, nausea/vomiting), several single-symptom items (dyspnea, insomnia, appetite loss, constipation, diarrhea, financial difficulties), and a Global Health Status/Quality of Life scale, reflecting experiences during the past week [30,31]. Scores were transformed to a 0–100 scale according to the scoring manual, with higher scores on functional scales and Global Health Status indicating better functioning and quality of life, and higher symptom scores indicating greater severity. Data were analyzed using IBM SPSS Statistics (Version 27.0).

### 2.5. Study analysis

Data were systematically coded and analyzed using IBM SPSS Statistics version 27.0. This cross-sectional study included adult cancer patients receiving chemotherapy and aimed to identify factors associated with Global Health Status/Quality of Life (QoL) and medication burden. Two multiple linear regression models were performed: the first model assessed factors associated with Global Health Status/QoL, while the second model examined factors associated with the total number of non-oncological medications used by patients.

The independent variables included in the regression models were age group, gender, marital status, occupation, education level, residence, monthly income, comorbidity status, duration since cancer diagnosis, and excessive polypharmacy status. In the QoL regression model, excessive polypharmacy (≥10 non-oncological medications) was included as a categorical variable (yes/no) to assess its association with Global Health Status/QoL. In the medication burden regression model, the outcome was analyzed as the continuous number of non-oncological medications used by each patient. Medication burden included supportive and concomitant therapies, while systemic anti-cancer agents were excluded to avoid confounding related to chemotherapy treatment regimens.

Initial analyses involved descriptive statistics to characterize the study population. Continuous variables were presented as means with standard deviations, whereas categorical variables were summarized using frequencies and percentages. These analyses were used to describe sociodemographic characteristics, clinical features, cancer-related variables, medication burden, and quality of life outcomes.

Quality of life was assessed using the Arabic version of the European Organization for Research and Treatment of Cancer Quality of Life Questionnaire-Core 30 (EORTC QLQ-C30) [30]. Scores were calculated according to the EORTC scoring manual and transformed to a standardized 0–100 scale. Higher scores on functional scales and Global Health Status/QoL represented better functioning and overall quality of life, whereas higher scores on symptom scales indicated greater symptom severity.

Polypharmacy was defined as the concurrent use of five or more (≥5) non-oncological medications, including supportive and concomitant therapies. Excessive polypharmacy was defined as the use of ten or more (≥10) non-oncological medications. Because nearly all participants met the criterion for polypharmacy (≥5 medications), this threshold showed limited variability and was not suitable for regression analysis. Therefore, medication burden was analyzed as a continuous variable representing the total number of non-oncological medications. Differences in Global Health Status/QoL scores according to excessive polypharmacy status were explored using independent-samples t-tests.

Multiple linear regression analyses were conducted to identify independent factors associated with Global Health Status/QoL and the total number of non-oncological medications. Variables with substantial missing information were not included in multivariable analyses. Specifically, cancer stage was excluded because data were available for only 151 participants (83 missing values), which would have substantially reduced the analytical sample size and statistical power. The remaining predictor variables had minimal missing data, and the regression models were based on 234 participants.

Regression diagnostics were performed to evaluate the assumptions of linearity, normal distribution of residuals, homoscedasticity, and absence of multicollinearity, and these assumptions were considered adequately satisfied. A two-sided p-value of <0.05 was considered statistically significant throughout all analyses.

## 3. Results

### 3.1. Sociodemographic and clinical characteristics

Table 1 presents the sociodemographic and selected clinical characteristics of the participants (N = 234). Among participants with available age data, the largest age group was 31–50 years (109/229; 47.6%), followed by ≥51 years (79/229; 34.5%) and 18–30 years (41/229; 17.9%). Females comprised 148/234 (63.2%) of the sample, while males represented 86/234 (36.8%). Most participants were married (178/234; 76.1%) and unemployed (195/234; 83.3%). Among participants with available education data, 117/233 (50.2%) were literate and 116/233 (49.8%) were illiterate. Among participants with available residence data, 144/233 (61.8%) resided in rural areas. Most participants reported a monthly income ≤80,000 YER (206/234; 88.0%). Comorbidities were reported by 47/234 (20.1%) participants. Comorbidities were reported by 47/234 (20.1%) participants, with hypertension (25/234; 10.7%), diabetes mellitus (14/234; 6.0%), and heart disease (11/234; 4.7%) being the most frequently reported conditions. Overall, the cohort was predominantly female, married, rural-residing, and of lower socioeconomic status.

**Table 1:** Sociodemographic and clinical characteristics of participants (n = 234).

Variable	Category	n (%)
**Age (years)** †	18–30	41 (17.9)
	31–50	109 (47.6)
	≥51	79 (34.5)
**Gender**	Male	86 (36.8)
	Female	148 (63.2)
**Marital status**	Married	178 (76.1)
	Non-married	56 (23.9)
**Occupation**	Employed	39 (16.7)
	Unemployed	195 (83.3)
**Education** ‡	Literate	117 (50.2)
	Illiterate	116 (49.8)
**Residence** §	Urban	89 (38.2)
	Rural	144 (61.8)
**Monthly income (YER)**	≤80,000	206 (88.0)
	>80,000	28 (12.0)
**Comorbidities**	Yes	47 (20.1)
	No	187 (79.9)
	**Specific comorbid conditions** *	
	Hypertension	25 (10.7)
	Diabetes mellitus	14 (6.0)
	Heart disease	11 (4.7)
	Kidney disease	1 (0.4)
	Other diseases∞	9 (3.8)
**Cancer stage¶**	Early stage (I–II)	21 (13.9)
	Advanced stage (III–IV)	130 (86.1)
**Cancer type**	Breast	23 (9.8)
	Colorectal	54 (23.1)
	Oral	29 (12.4)
	Gynecologic	15 (6.4)
	Lung	6 (2.6)
	Hodgkin lymphoma	4 (1.7)
	Nasopharyngeal	16 (6.8)
	Gastrointestinal and esophageal	32 (13.7)
	Pancreatic	6 (2.6)
	Other	49 (20.9)
**Time since diagnosis** #	≤1 year	162 (71.1)
	>1 year	66 (28.9)

^†^ Age data available for 229 participants (5 missing).

^‡^ Education data available for 233 participants (1 missing).

^§^ Residence data available for 233 participants (1 missing).

^¶^ Cancer stage data available for 151 participants (83 missing).

^#^ Time since diagnosis data available for 228 participants (6 missing).

*Participants could have more than one comorbid condition; therefore, percentages for specific comorbidities were calculated using the total study population (n = 234) and may not sum to the total number of participants with comorbidities. ∞ Other diseases included benign prostatic hyperplasia, thyroid disorders, fatty liver disease, HIV infection, peptic ulcer disease, and other reported conditions.

### 3.2. Cancer-Related Characteristics

Cancer types and duration since diagnosis are summarized in Table 1. Colorectal cancer was the most frequent diagnosis (23.1%, n = 54/234), followed by other cancers (20.9%, n = 49/234) and gastrointestinal and esophageal cancers (13.7%, n = 32/234). Breast (9.8%, n = 23/234), oral (12.4%, n = 29/234), and nasopharyngeal cancers (6.8%, n = 16/234) were also reported, with smaller proportions for gynecologic (6.4%, n = 15/234), lung (2.6%, n = 6/234), pancreatic (2.6%, n = 6/234), and Hodgkin’s lymphoma (1.7%, n = 4/234). Regarding cancer stage, most participants with available stage data had advanced disease (III–IV) (86.1%, n = 130/151), compared with those with early-stage disease (I–II) (13.9%, n = 21/151), while 83 participants (35.5%, n = 83/234) had missing stage data. In terms of disease duration, the majority of participants had been diagnosed within the past year (71.1%, n = 162/228). Overall, these findings highlight the predominance of colorectal and gastrointestinal cancers in this study, along with a higher proportion of advanced-stage disease among participants with available stage data.

### 3.3. Medication Use

Table 2 summarizes the use of systemic anti-cancer medications, supportive medications, and natural products/herbal and traditional remedies. The most commonly used anti-cancer medications were 5-Fluorouracil (62.8%, n = 147/234), Leucovorin (37.2%, n = 87/234), and Oxaliplatin and Cisplatin (32.5% each, n = 76/234 each). Less frequently used drugs included Doxorubicin (12.5%, n = 29/234), Cyclophosphamide (11.1%, n = 26/234), Paclitaxel (8.5%, n = 20/234), and Etoposide (6.8%, n = 16/234). Supportive medications were widely prescribed, especially Pantoprazole (99.1%, n = 232/234), Dexamethasone (95.3%, n = 223/234), and Ondansetron (94.9%, n = 222/234). Use of natural products/herbal and traditional remedies was limited, with Caper bush (15.0%, n = 35/234) and Honey (9.4%, n = 22/234) being the most commonly reported.

**Table 2:** Summary of systemic anti-cancer, supportive, and natural products / Herbal and traditional remedies use, and medication burden (n = 234).

Medication / Therapy	Yes, n (%)	No, n (%)
5-Fluorouracil (5-FU)	147 (62.8)	87 (37.2)
Oxaliplatin	76 (32.5)	158 (67.5)
Leucovorin (Folinic acid)	87 (37.2)	147 (62.8)
Avastin	23 (9.8)	211 (90.2)
Irinotecan	14 (6.0)	220 (94.0)
Cisplatin	76 (32.5)	158 (67.5)
Doxorubicin*	29 (12.5)	203 (87.5)
Cyclophosphamide	26 (11.1)	208 (88.9)
Rituximab	8 (3.4)	226 (96.6)
Paclitaxel	20 (8.5)	214 (91.5)
Cytarabine	5 (2.1)	229 (97.9)
Carboplatin	7 (3.0)	227 (97.0)
Docetaxel	9 (3.8)	225 (96.2)
Methotrexate	12 (5.1)	222 (94.9)
Vincristine	11 (4.7)	223 (95.3)
Ifosfamide	11 (4.7)	223 (95.3)
Etoposide	16 (6.8)	218 (93.2)
Mesna	13 (5.6)	221 (94.4)
**Supportive Medications**		
Pantoprazole	232 (99.1)	2 (0.9)
Dexamethasone	223 (95.3)	11 (4.7)
Ondansetron	222 (94.9)	12 (5.1)
Paracetamol (SOS)	185 (79.1)	49 (20.9)
Metoclopramide	184 (78.6)	50 (21.4)
MgSO_4_	174 (74.4)	60 (25.6)
Pheniramine	163 (69.7)	71 (30.3)
Furosemide (SOS)	158 (67.5)	76 (32.5)
Tramadol (SOS)	114 (48.7)	120 (51.3)
KCl	112 (47.9)	122 (52.1)
Vitamin C	98 (41.9)	136 (58.1)
Vitamin B	97 (41.5)	137 (58.5)
Ceftriaxone	16 (6.8)	218 (93.2)
Metformin	12 (5.1)	222 (94.9)
Mannitol	10 (4.3)	224 (95.7)
**Natural Products / Herbal and Traditional Remedies**		
Caper bush	35 (15.0)	199 (85.0)
Honey	22 (9.4)	212 (90.6)
Black Seed	18 (7.7)	216 (92.3)
Garlic	17 (7.3)	217 (92.7)
Ginger	11 (4.7)	223 (95.3)
Liv52	11 (4.7)	223 (95.3)
Curcumin	10 (4.3)	224 (95.7)
**Medication Burden**		
≥5 Non-oncological medications	222 (94.9)	12 (5.1)
≥10 Non-oncological medications	132 (56.4)	102 (43.6)

*Doxorubicin valid N = 232 (2 missing cases); SOS (as-needed) medications were defined as drugs prescribed for intermittent use according to clinical need. These medications were included in the medication count when documented in medical records, regardless of actual administration frequency; Natural products and traditional remedies (e.g., honey, garlic, ginger, black seed, caper bush, Liv52, and curcumin) were recorded based on patient self-report and were included in the total medication count for polypharmacy assessment to reflect the overall therapeutic burden and potential for drug–herb interactions in this population.

### 3.4. Polypharmacy and medication burden

Participants were frequently exposed to polypharmacy (Table 2). Total medications per participant ranged from 5 to 22, with 77.0% receiving 2–3 cancer medications. Regarding non-oncological medications, 94.9% of participants used five or more drugs, and 56.4% used ten or more. These findings indicate a substantial medication burden, highlighting the potential for polypharmacy-related complications.

### 3.5. EORTC QLQ-C30 functional and symptom domains

Descriptive statistics for the EORTC QLQ-C30 scales are presented in Table 3. Among the functional scales, cognitive functioning showed the highest scores (median = 83.33), followed by social functioning (median = 66.67) and emotional functioning (median = 41.67). Physical functioning demonstrated moderate levels (median = 46.67), while role functioning was the lowest (median = 16.67), indicating substantial limitations in daily activities among participants.

**Table 3:** Descriptive statistics of EORTC QLQ-C30 scales (n = 234).

Variable	Mean ± SD	Median (IQR)
Physical Functioning	48.15 ± 25.11	46.67 (40.00)
Role Functioning	32.27 ± 36.99	16.67 (66.67)
Emotional Functioning	50.71 ± 34.75	41.67 (58.33)
Cognitive Functioning	69.94 ± 31.25	83.33 (50.00)
Social Functioning	62.75 ± 35.65	66.67 (66.67)
Fatigue	61.06 ± 29.68	66.67 (55.56)
Nausea/Vomiting	35.47 ± 33.73	33.33 (66.67)
Pain	59.62 ± 32.30	66.67 (50.00)
Dyspnea	36.75 ± 37.67	33.33 (66.67)
Insomnia	54.13 ± 38.63	66.67 (100.00)
Appetite Loss	54.42 ± 36.83	66.67 (66.67)
Constipation	34.47 ± 39.29	33.33 (66.67)
Diarrhea	28.35 ± 36.39	0.00 (66.67)
Financial Difficulties	82.34 ± 27.30	100.00 (33.33)
Global Health Status / QoL	47.86 ± 26.67	50.00 (33.33)

Data are presented as mean ± standard deviation and median (interquartile range). All scales are scored from 0 to 100. Higher scores on functional scales and Global Health Status indicate better functioning, whereas higher scores on symptom scales indicate greater symptom burden.

Regarding symptom scales, financial difficulties were the most severe (median = 100.00), followed by fatigue (median = 66.67), pain (median = 66.67), appetite loss (median = 66.67), and insomnia (median = 66.67). Other symptoms, including nausea/vomiting, dyspnea, constipation, and diarrhea, showed comparatively lower median scores, although variability was observed across patients. The median Global Health Status/Quality of Life score was 50.00, indicating a moderate overall perceived quality of life among participants.

### 3.6. Effect of polypharmacy on global health status/QoL

Table 4 presents the comparison of QoL by excessive polypharmacy (≥10 non-oncological medications). Participants with excessive polypharmacy (n = 132) had a mean QoL score of 46.40 ± 22.63, compared with 49.75 ± 31.15 for those taking <10 non-oncological medications (n = 102). The difference was not statistically significant (t = −0.92, df = 177.46, p = .361), with a small effect size (Cohen’s d = −0.13), indicating that excessive polypharmacy did not significantly affect global QoL.

**Table 4:** Comparison of global health status/QoL by excessive polypharmacy (≥10 Non-oncological Medications) (n = 234).

Polypharmacy (≥10 Non-oncological medications)	N	Mean ± SD	t	df	p	Mean Difference (95% CI)	Cohen's d
**Yes**	132	46.40 ± 22.63	−0.92	177.46	.361	−3.35 (−10.58, 3.87)	−0.13
**No**	102	49.75 ± 31.15					

SD = standard deviation; df = degrees of freedom; CI = confidence interval; Cohen’s d = standardized effect size.

### 3.7. Factors associated with non-oncological polypharmacy in cancer patients

A multiple linear regression analysis was conducted to examine sociodemographic and clinical factors associated with the number of non-oncological medications among cancer patients, reflecting polypharmacy (Table 5). The predictors included age group, gender, marital status, occupation, education level, residence, monthly income, comorbidity status, and time since diagnosis.

**Table 5:** Multiple linear regression analysis of factors associated with the number of non-oncological medications (n = 234).

Independent variable	B	SE	β	t	p-value	95% CI for B
**Constant**	8.706	0.846	—	10.290	<0.001	7.039, 10.374
**Age (31–50)**	-0.052	0.567	-0.008	-0.092	0.926	-1.169, 1.064
**Age (≥51)**	-0.182	0.630	-0.028	-0.288	0.773	-1.424, 1.060
**Gender (Female)**	1.176	0.469	0.183	2.506	0.013*	0.251, 2.101
**Marital status (Non-married)**	-0.862	0.491	-0.118	-1.754	0.081	-1.829, 0.106
**Occupation (Unemployed)**	0.847	0.591	0.102	1.432	0.153	-0.318, 2.012
**Education (Illiterate)**	0.392	0.515	0.063	0.761	0.447	-0.623, 1.407
**Residence (Rural residence)**	-0.068	0.446	-0.011	-0.152	0.879	-0.947, 0.811
**Income (>80,000 YER)**	0.801	0.620	0.084	1.293	0.197	-0.420, 2.022
**Comorbidity (No)**	-0.417	0.512	-0.054	-0.815	0.416	-1.425, 0.591
**Time since diagnosis (>1 year)**	0.421	0.446	0.061	0.943	0.347	-0.458, 1.300

R = 0.310, R^2^ = 0.096, Adjusted R^2^ = 0.055, F(10, 223) = 2.367, p = .011, Std. Error of the Estimate = 3.03; *Significant predictor (p < 0.05). *Reference categories: age 18–30 years, male gender, married, employed, literate, urban residence, monthly income ≤80,000 YER, presence of comorbidity, time since diagnosis ≤1 year,*

The overall model was statistically significant, F(10, 223) = 2.367, p = .011, explaining 9.6% of the variance in non-oncological medication use (R^2^ = 0.096; Adjusted R^2^ = 0.055), with a standard error of the estimate of 3.03.

Among all included variables, female gender was the only statistically significant predictor of higher non-oncological medication use. Female patients had, on average, 1.18 more medications compared with males (B = 1.176, SE = 0.469, β = 0.183, t = 2.506, p = .013, 95% CI: 0.251 to 2.101).

All other variables—including age group, marital status, occupation, education level, residence, monthly income, comorbidity status, and duration of illness—were not statistically significant predictors in the adjusted model (all p > .05).

These findings suggest that gender may play a role in non-oncological medication burden among cancer patients, whereas other sociodemographic and clinical characteristics showed no independent association with polypharmacy in this cohort.

Assumptions of linear regression were assessed using visual inspection of standardized residuals (histogram and normal P–P plot), which indicated an approximately normal distribution of residuals, with standardized residuals ranging from -2.33 to 2.81, suggesting that the normality assumption was adequately met.

### 3.8. Factors associated with global health status / QoL

A multiple linear regression analysis was conducted to examine sociodemographic, clinical, and medication-related factors associated with Global Health Status/Quality of Life (QoL) among cancer patients (Table 6). The predictors included age group, gender, marital status, occupation, education level, residence, monthly income, comorbidity status, time since diagnosis, and presence of excessive polypharmacy (≥10 non-oncological medications).

**Table 6:** Multiple linear regression analysis of factors associated with global health status/QoL (0–100) (n = 234).

Independent variable	B	SE	β	t	p-value	95% CI for B
**Constant**	60.62	7.63	—	7.95	<0.001	45.59, 75.65
**Age (31–50)**	-1.83	4.80	-0.03	-0.38	0.704	-11.30, 7.64
**Age (≥51)**	-9.74	5.34	-0.17	-1.82	0.070	-20.26, 0.79
**Gender (Female)**	-10.13	4.09	-0.18	-2.48	0.014*	-18.19, -2.07
**Marital status (Non-married)**	-3.54	4.16	-0.06	-0.85	0.396	-11.75, 4.66
**Occupation (Unemployed)**	4.86	5.01	0.07	0.97	0.332	-5.00, 14.73
**Education (Illiterate)**	-8.35	4.39	-0.16	-1.90	0.058	-16.99, 0.29
**Residence (Rural residence)**	-3.31	3.79	-0.06	-0.87	0.384	-10.77, 4.16
**Income (>80,000 YER)**	6.36	5.25	0.08	1.21	0.227	-3.98, 16.70
**Comorbidity (No)**	1.46	4.33	0.02	0.34	0.736	-7.07, 9.99
**Time since diagnosis (>1 year)**	-1.79	3.79	-0.03	-0.47	0.636	-9.25, 5.67
**Excessive polypharmacy (≥10 medications)**	-1.52	3.63	-0.03	-0.42	0.675	-8.68, 5.64

R = 0.349; R^2^ = 0.122; Adjusted R^2^ = 0.078; F(11,222) = 2.803; p = 0.002; Standard error of the estimate = 25.60; *Significant predictor (p < 0.05). *Reference categories: age 18–30 years, male gender, married, employed, literate, urban residence, monthly income ≤80,000 YER, presence of comorbidity, time since diagnosis ≤1 year, and excessive polypharmacy <10 medications.*

The overall model was statistically significant, F(11, 222) = 2.803, p = .002, explaining 12.2% of the variance in QoL scores (R^2^ = 0.122; Adjusted R^2^ = 0.078), with a standard error of the estimate of 25.60.

Among all variables, female gender was significantly associated with lower QoL scores, with females reporting a 10.13-point reduction compared with males (B = -10.130, SE = 4.092, β = -0.184, t = -2.475, p = .014, 95% CI: -18.194 to -2.065).

In addition, age ≥ 51 years showed a borderline negative association with QoL (B = -9.735, p = .070), and illiteracy also approached statistical significance (B = -8.351, p = .058). However, all other variables—including age < 50 years, marital status, occupation, residence, monthly income, comorbidity status, time since diagnosis, and excessive polypharmacy—were not statistically significant predictors of QoL (all p > .05).

These findings indicate that female cancer patients experienced significantly poorer quality of life, while other sociodemographic, clinical, and medication-related factors, including polypharmacy, were not independently associated with QoL in the adjusted model.

Diagnostic checks for regression assumptions indicated that standardized residuals were normally distributed (range: -2.43 to 2.33), with no evidence of severe outliers or violations of model assumptions.

## 4. Discussion

This study found a very high prevalence of polypharmacy among cancer patients receiving chemotherapy in Yemen, with 94.9% using ≥5 non-oncological medications and 56.4% exposed to excessive polypharmacy (≥10 medications). Despite the substantial medication burden, excessive polypharmacy was not significantly associated with global quality of life (QoL). The mean QoL score (47.9 ± 26.7) indicated moderate well-being, with high levels of financial difficulties, fatigue, and pain. Female gender was the only significant independent predictor of poorer QoL and was also associated with higher non-oncological medication use. Overall, these findings suggest that sociodemographic factors may have a greater influence on QoL than medication burden alone.

The prevalence of polypharmacy defined as the use of five or more medications observed in the present study (94.9%) is higher than that reported in many oncology populations worldwide. Studies from developed countries have generally documented lower prevalence estimates. For example, a study among older cancer patients receiving palliative chemotherapy in South Korea reported a polypharmacy prevalence of 45.2% [32]. Similarly, a systematic review and meta-analysis of older lung cancer patients reported a pooled polypharmacy prevalence of approximately 35% [33]. Other studies conducted in Europe and North America have reported prevalence estimates ranging from 38% to 75.8% among older adults with cancer [34–37].

In contrast, studies from the Middle East and other developing regions have reported relatively higher rates. A cross-sectional study among ambulatory cancer patients in Saudi Arabia found that 79% of patients experienced polypharmacy [38]. Likewise, research conducted among elderly cancer patients in Jordan reported that 83% of patients were prescribed five or more medications [39]. An outpatient oncology study from Turkey reported an even higher prevalence, with 94.7% of elderly cancer patients experiencing polypharmacy [40]. Additionally, a recent multicenter study in Ethiopian oncology centers reported a polypharmacy prevalence of 70.2% among older adults with cancer [41].

These variations across studies may reflect differences in patient characteristics, healthcare systems, study populations, and definitions of polypharmacy. This elevated prevalence may be attributed to the complexity of chemotherapy protocols, which often require multiple supportive medications such as antiemetics, corticosteroids, gastric protectants, and analgesics. In addition, limited access to comprehensive medication review services in resource-constrained settings may contribute to the accumulation of medications over time. While the high prevalence of polypharmacy highlights the complexity of pharmacotherapy in oncology care, understanding its potential impact on patients’ quality of life remains equally important.

The mean Global Health Status/Quality of Life (QoL) score observed in this study (47.86) indicates a moderate level of perceived health-related quality of life among patients undergoing chemotherapy. Similar patterns have been reported in oncology populations where cancer treatment is associated with substantial symptom burden and functional limitations. Studies using the EORTC QLQ-C30 have consistently shown that fatigue, pain, and impaired physical functioning are among the most prominent factors contributing to reduced quality of life during chemotherapy. For example, a study among chemotherapy patients in Pakistan reported significant associations between metastatic disease and higher levels of fatigue, pain, and financial difficulties [42].Likewise, research among breast cancer patients undergoing chemotherapy in India found considerable impairments in physical, social, and cognitive functioning accompanied by elevated symptom burden, including fatigue and pain [43].

Financial difficulties represented the highest symptom score in the present study, highlighting the substantial economic burden associated with cancer care in Yemen. Similar findings have been reported in other low- and middle-income settings. For instance, a study conducted among breast cancer patients in Morocco identified financial difficulties as one of the most prominent concerns affecting patients’ quality of life [44]. In the Yemeni context, the absence of comprehensive health insurance coverage, high out-of-pocket expenditures, and transportation costs for patients traveling from rural areas may further exacerbate this burden.

Although polypharmacy is increasingly recognized as a common issue in oncology care, relatively few studies have specifically examined its relationship with health-related quality of life among cancer patients. Available evidence suggests that higher medication burden may negatively influence patient-reported outcomes. For example, a population-based study in the United States reported that cancer survivors experiencing polypharmacy had significantly lower physical health-related quality of life scores compared with those without polypharmacy [26]. Similarly, research among older adults with prostate cancer demonstrated that both polypharmacy and excessive polypharmacy were associated with significant declines in physical and mental QoL [27]. Findings from the Cancer and Aging Resilience Evaluation (CARE) registry also indicated that patients taking multiple medications were more likely to report functional limitations, frailty, and poorer QoL [45]. In addition, psychotropic polypharmacy has been linked to reduced physical and mental QoL among cancer survivors [28]. Despite these findings, the evidence remains limited, and further research is needed to better understand how medication burden influences quality of life among cancer patients in different clinical and socioeconomic contexts.

Interestingly, despite the relatively high prevalence of excessive polypharmacy (56.4%) observed in the present study, it was not independently associated with global health status or overall quality of life. This null finding should be interpreted cautiously, as the distribution of exposure may still have limited statistical contrast between groups, potentially reducing sensitivity to detect an association with QoL. This finding may reflect the complex clinical context of oncology care, where multiple medications are frequently prescribed as part of supportive management during chemotherapy. Many of these medications, including antiemetics, analgesics, and corticosteroids, are intended to control treatment-related adverse effects and alleviate symptoms, potentially mitigating the negative impact of increased medication burden on patients’ perceived well-being. Furthermore, quality of life in cancer patients is shaped by multiple interacting factors beyond medication use alone. Clinical characteristics such as disease stage, treatment-related toxicity, and symptom severity, as well as socioeconomic conditions, may exert a stronger influence on patients’ overall well-being. Consequently, medication count alone may not adequately capture the multifactorial determinants of quality of life among patients receiving cancer treatment. Therefore, the absence of a significant association should not be interpreted as evidence that polypharmacy has no effect on QoL but rather that such an effect may have been difficult to detect in this study.

In exploring factors that may contribute to increased medication burden, female gender was significantly associated with a higher number of non-oncological medications in this study, suggesting that women may be more exposed to polypharmacy during chemotherapy. This finding is consistent with previous research showing that female patients are more likely to use multiple medications compared with males, partly due to higher healthcare utilization, greater symptom reporting, and a higher prevalence of certain chronic conditions requiring pharmacological management [46,47].

Comorbidity status was not independently associated with the number of non-oncological medications in the present study. Although multimorbidity has been reported as an important contributor to polypharmacy, our findings did not demonstrate a statistically significant association between comorbidity status and medication burden. This may be explained by the high medication requirements related to cancer treatment and supportive care, which may contribute to medication burden regardless of patients’ baseline comorbid conditions. Nevertheless, patients with multiple chronic conditions often require additional long-term medications alongside cancer therapy, potentially increasing the risk of drug–drug interactions and treatment complexity [48]. These findings highlight the importance of comprehensive medication review strategies for all cancer patients, with particular attention to those with complex treatment regimens, to optimize medication safety and minimize unnecessary medication burden.

Beyond medication burden, gender differences were also observed in patient-reported outcomes. Female patients in this study reported significantly lower quality of life compared with males, a finding that is consistent with evidence from other cancer populations. Several studies have found that women with cancer experience higher symptom burden and poorer health‑related quality of life than their male counterparts. For example, research examining gender differences in quality of life among cancer patients with pain showed that female patients reported significantly lower psychological quality of life than male patients, with symptoms such as fatigue and depression more strongly affecting overall well‑being in women [49]. In a study of colorectal cancer patients, women reported higher levels of fatigue, pain, and impaired physical and social functioning compared with men, and gender was identified as an independent predictor of poorer functional scores [50]. Similarly, assessments of quality of life have demonstrated that poorer functional status and higher symptom severity in female patients are linked with reduced overall QoL scores [51].

In Yemen, these disparities may be amplified by structural and sociocultural factors, including limited healthcare access, reduced financial autonomy, and constrained social support. Such barriers may delay care-seeking, limit access to medications, and reduce autonomy in treatment-related decision-making, ultimately contributing to poorer perceived quality of life among female patients [52].

Beyond patient-level disparities, these findings have important practical implications for oncology care delivery in resource-limited and conflict-affected settings. Evidence from low- and middle-income countries suggests that structured medication review programs, integration of clinical pharmacists into multidisciplinary oncology teams, and context-adapted deprescribing strategies can reduce inappropriate medication use and improve patient safety and quality of life [53,54]. However, implementation in fragile health systems remains challenging due to limited human resources and overburdened oncology services. Adaptation of these approaches to the Yemeni context may therefore offer a feasible strategy to optimize pharmacotherapy, reduce unnecessary medication use, and support symptom management in cancer patients.

Despite the lack of a direct association between polypharmacy and QoL in the present study, the high medication burden highlights the importance of structured medication review and optimization strategies in oncology practice. Integration of clinical pharmacists into multidisciplinary cancer care teams, along with structured medication reconciliation and deprescribing approaches tailored to resource-limited settings, may help reduce inappropriate medication use, prevent drug–drug interactions, and improve treatment safety. In addition, interventions targeting symptom control and financial support may further enhance quality of life among cancer patients in Yemen.

This study has several limitations that should be considered when interpreting the findings. First, the cross-sectional design limits the ability to determine causal relationships between polypharmacy and quality of life. Second, the study was conducted in a single major tertiary referral oncology centre, which may restrict the generalizability of the results to other healthcare settings or cancer populations within Yemen, particularly those in regions with different levels of healthcare infrastructure, access to oncology services, and socioeconomic conditions. Third, although medication information was verified through medical records when possible, some data relied on patient self-report, which may be subject to recall bias, particularly for over-the-counter or Natural Products / Herbal and Traditional Remedies. Fourth, polypharmacy was assessed based on the number of medications without evaluating medication appropriateness, potential drug–drug interactions, or adherence, all of which may influence health-related quality of life. In addition, the analysis of excessive polypharmacy (≥10 medications) may have been affected by limited variability in exposure distribution, which could reduce statistical contrast and the ability to detect modest associations with quality of life.

Furthermore, information on treatment intensity and other clinically relevant factors, such as performance status and detailed treatment-related characteristics, was not available and therefore could not be included in the analyses. In addition, cancer stage was not included in the regression models because of incomplete clinical documentation, which resulted in substantial missing data. These unmeasured factors may have influenced medication burden and quality of life and should be considered in future studies.

Finally, although sample size adequacy was assessed using Green’s rule of thumb for multiple regression, a formal a priori power calculation based on an expected effect size, alpha level, and desired statistical power was not performed before data collection. Future studies should incorporate prospective power calculations and include additional clinically relevant variables to strengthen the evaluation of factors associated with medication burden and quality of life.

Nevertheless, this study provides important preliminary evidence on the prevalence of polypharmacy and its relationship with health-related quality of life among cancer patients undergoing chemotherapy in Yemen. It contributes valuable baseline data to a relatively underexplored area of oncology research in low-resource settings and may help inform future studies, clinical practice, and policy initiatives aimed at optimizing medication management and improving the quality of life of cancer patients.

## 5. Conclusion

This study demonstrated a high prevalence of non-oncological polypharmacy among cancer patients receiving chemotherapy in Yemen, while excessive polypharmacy was not independently associated with overall quality of life. Female gender was associated with both greater medication burden and poorer quality of life, highlighting the influence of sociodemographic factors on patient well-being. These findings underscore the importance of multidisciplinary supportive care, including medication review and symptom management, and provide baseline evidence to inform future research and oncology care in resource-limited settings.

## Supporting information

S1 Data Dataset supporting the findings of the study. The file contains the de-identified data used for the analysis of polypharmacy and quality of life among Yemeni cancer patients undergoing chemotherapy.
(XLSX)

## References

[1] Bray F, Laversanne M, Weiderpass E, Soerjomataram I. The ever-increasing importance of cancer as a leading cause of premature death worldwide. Cancer. 2021;127(16):3029–30. doi: 10.1002/cncr.33587 34086348

[2] Sung H, Ferlay J, Siegel RL, Laversanne M, Soerjomataram I, Jemal A. Global cancer statistics 2020: GLOBOCAN estimates of incidence and mortality worldwide for 36 cancers in 185 countries. CA Cancer J Clin. 2021;71:209–49. doi: 10.3322/caac.21660 33538338

[3] Bray F, Laversanne M, Sung H, Ferlay J, Siegel RL, Soerjomataram I. Global cancer statistics 2022: GLOBOCAN estimates of incidence and mortality worldwide for 36 cancers in 185 countries. CA Cancer J Clin. 2024;74:229–63. doi: 10.3322/caac.21834 38572751

[4] Ibrahim A, El Baldi M, Mohammed S, El Rhazi K, Benazzouz B. Cancer statistics in Yemen: incidence and mortality, in 2020. BMC Public Health. 2024;24:962. doi: 10.1186/s12889-024-18207-4 PMC10996160 38580993

[5] Ferlay JEMLFLMCMMLPMZASIBF. Global Cancer Observatory: Cancer Today. Lyon, France: International Agency for Research on Cancer. 2024. https://gco.iarc.who.int/today

[6] Abbas Z, Rehman S. An overview of cancer treatment modalities. Neoplasm. InTech. 2018. 10.5772/intechopen.76558

[7] Donnelly JG. Pharmacogenetics in Cancer Chemotherapy. Therapeutic Drug Monitoring. 2004;26(2):231–5. doi: 10.1097/00007691-200404000-00026 15228171

[8] Pearce A, Haas M, Viney R, Pearson S-A, Haywood P, Brown C, et al. Incidence and severity of self-reported chemotherapy side effects in routine care: A prospective cohort study. PLoS One. 2017;12(10):e0184360. doi: 10.1371/journal.pone.0184360 29016607 PMC5634543

[9] Mustian KM, Darling TV, Janelsins MC, Jean-Pierre P, Roscoe JA, Morrow GR. Chemotherapy-Induced Nausea and Vomiting. US Oncol. 2008;4(1):19–23. doi: 10.17925/ohr.2008.04.1.19 24761161 PMC3994039

[10] Hwang SS, Chang VT, Cogswell J, Kasimis BS. Clinical relevance of fatigue levels in cancer patients at a Veterans Administration Medical Center. Cancer. 2002;94(9):2481–9. doi: 10.1002/cncr.10507 12015774

[11] Smith KS, Hoogland AI, Welniak T, Nguyen OL, Rodriguez Y, Li X, et al. Acute cancer-related symptoms and concerns among patients receiving chemotherapy: current state of the science. Support Care Cancer. 2024;32(3):184. doi: 10.1007/s00520-024-08385-7 38393418 PMC11273955

[12] Maggiore RJ, Dale W, Gross CP, Feng T, Tew WP, Mohile SG, et al. Polypharmacy and potentially inappropriate medication use in older adults with cancer undergoing chemotherapy: effect on chemotherapy-related toxicity and hospitalization during treatment. J Am Geriatr Soc. 2014;62(8):1505–12. doi: 10.1111/jgs.12942 25041361 PMC4134360

[13] Maher RL, Hanlon J, Hajjar ER. Clinical consequences of polypharmacy in elderly. Expert Opin Drug Saf. 2014;13(1):57–65. doi: 10.1517/14740338.2013.827660 24073682 PMC3864987

[14] Maggiore RJ, Gross CP, Hurria A. Polypharmacy in older adults with cancer. Oncologist. 2010;15(5):507–22. doi: 10.1634/theoncologist.2009-0290 20418534 PMC3227983

[15] World Health Organization. Patient Safety: Medication Safety in Polypharmacy. https://www.who.int/docs/default-source/patient-safety/who-uhc-sds-2019-11-eng.pdf. 2019. Accessed 2026 March 15.

[16] Varghese D, Patel P. Polypharmacy. StatPearls. Treasure Island (FL): StatPearls Publishing. 2026.

[17] Ramsdale E, Mohamed M, Yu V, Otto E, Juba K, Awad H. Polypharmacy, potentially inappropriate medications, and drug-drug interactions in vulnerable older adults with advanced cancer initiating cancer treatment. Oncologist. 2022;27:e580-8. doi: 10.1093/oncolo/oyac053 PMC9255971 35348764

[18] Betts AC, Murphy CC, Shay LA, Balasubramanian BA, Markham C, Allicock M. Polypharmacy and prescription medication use in a population-based sample of adolescent and young adult cancer survivors. J Cancer Surviv. 2023;17(4):1149–60. doi: 10.1007/s11764-021-01161-0 34997910 PMC10614319

[19] Murphy CC, Fullington HM, Alvarez CA, Betts AC, Lee SJC, Haggstrom DA, et al. Polypharmacy and patterns of prescription medication use among cancer survivors. Cancer. 2018;124(13):2850–7. doi: 10.1002/cncr.31389 29645083 PMC6147245

[20] Eyigor S, Kutsal YG, Toraman F, Durmus B, Gokkaya KO, Aydeniz A, et al. Polypharmacy, Physical and Nutritional Status, and Depression in the Elderly: Do Polypharmacy Deserve Some Credits in These Problems?. Exp Aging Res. 2021;47(1):79–91. doi: 10.1080/0361073X.2020.1846949 33183169

[21] Shah BM, Hajjar ER. Polypharmacy, Adverse Drug Reactions, and Geriatric Syndromes. Clin Geriatr Med. 2012;28:173–86. doi: 10.1016/j.cger.2012.01.002 22500537

[22] Annunziata MA, Muzzatti B, Flaiban C, Gipponi K, Carnaghi C, Tralongo P, et al. Long-term quality of life profile in oncology: a comparison between cancer survivors and the general population. Support Care Cancer. 2018;26(2):651–6. doi: 10.1007/s00520-017-3880-8 28918552

[23] Moffat P. Quality of life in patients with cancer. Int J Palliat Nurs. 2008;14(1):4. doi: 10.12968/ijpn.2008.14.1.28147 18414326

[24] Baltussen JC, de Glas NA, van Holstein Y, van der Elst M, Trompet S, Uit den Boogaard A, et al. Chemotherapy-Related Toxic Effects and Quality of Life and Physical Functioning in Older Patients. JAMA Netw Open. 2023;6(10):e2339116. doi: 10.1001/jamanetworkopen.2023.39116 37870832 PMC10594146

[25] Springer F, Matsuoka A, Obama K, Mehnert-Theuerkauf A, Uchitomi Y, Fujimori M. Quality of life in older patients with cancer and related unmet needs: A scoping review. Acta Oncol. 2025;64:516–26. doi: 10.2340/1651-226X.2025.42602 40230321 PMC12012652

[26] Babcock ZR, Kogut SJ, Vyas A. Association between polypharmacy and health-related quality of life among cancer survivors in the United States. J Cancer Surviv. 2020;14(1):89–99. doi: 10.1007/s11764-019-00837-y 31792811

[27] Yue Z, Xue X, Qian J. The association between polypharmacy and health-related quality of life among older adults with prostate cancer. J Geriatr Oncol. 2024;15(5):101772. doi: 10.1016/j.jgo.2024.101772 38676976

[28] Vyas A, Alghaith G, Hufstader-Gabriel M. Psychotropic polypharmacy and its association with health-related quality of life among cancer survivors in the USA: a population-level analysis. Qual Life Res. 2020;29(8):2029–37. doi: 10.1007/s11136-020-02478-6 32207028

[29] Green SB. How Many Subjects Does It Take To Do A Regression Analysis. Multivariate Behav Res. 1991;26(3):499–510. doi: 10.1207/s15327906mbr2603_7 26776715

[30] Aaronson NK, Ahmedzai S, Bergman B, Bullinger M, Cull A, Duez NJ, et al. The European organization for research and treatment of cancer QLQ-C30: A quality-of-life instrument for use in international clinical trials in oncology. J Natl Cancer Inst. 1993;85(5):365–76. doi: 10.1093/jnci/85.5.365 8433390

[31] Huijer HA-S, Sagherian K, Tamim H. Validation of the Arabic version of the EORTC quality of life questionnaire among cancer patients in Lebanon. Qual Life Res. 2013;22(6):1473–81. doi: 10.1007/s11136-012-0261-0 22961075

[32] Hong S, Lee JH, Chun EK, Kim KI, Kim JW, Kim SH. Polypharmacy, inappropriate medication use, and drug interactions in older Korean patients with cancer receiving first-line palliative chemotherapy. Oncologist. 2020;25:e502-11. doi: 10.1634/theoncologist.2019-0085 PMC7066695 32162799

[33] Tian F, Chen Z, Zhou D, Mo L. Prevalence of polypharmacy and potentially inappropriate medication use in older lung cancer patients: A systematic review and meta-analysis. Front Pharmacol. 2022;13:1044885. doi: 10.3389/fphar.2022.1044885 36588688 PMC9800788

[34] Leger DY, Moreau S, Signol N, Fargeas J-B, Picat M-A, Penot A, et al. Polypharmacy, potentially inappropriate medications and drug-drug interactions in geriatric patients with hematologic malignancy: Observational single-center study of 122 patients. J Geriatr Oncol. 2018;9(1):60–7. doi: 10.1016/j.jgo.2017.07.015 28851511

[35] Ramsdale E, Mohamed M, Yu V, Otto E, Juba K, Awad H. Polypharmacy, potentially inappropriate medications, and drug-drug interactions in vulnerable older adults with advanced cancer initiating cancer treatment. Oncologist. 2022;27:e580-8. doi: 10.1093/oncolo/oyac053 PMC9255971 35348764

[36] Elliot K, Tooze JA, Geller R, Powell BL, Pardee TS, Ritchie E, et al. The prognostic importance of polypharmacy in older adults treated for acute myelogenous leukemia (AML). Leuk Res. 2014;38(10):1184–90. doi: 10.1016/j.leukres.2014.06.018 25127690 PMC4182134

[37] Iurlo A, Nobili A, Latagliata R, Bucelli C, Castagnetti F, Breccia M, et al. Imatinib and polypharmacy in very old patients with chronic myeloid leukemia: effects on response rate, toxicity and outcome. Oncotarget. 2016;7(48):80083–90. doi: 10.18632/oncotarget.11657 27579540 PMC5346773

[38] Alwhaibi M, AlRuthia Y, Alhawassi TM, Almalag H, Alsalloum H, Balkhi B. Polypharmacy and comorbidities among ambulatory cancer patients: A cross-sectional retrospective study. J Oncol Pharm Pract. 2020;26(5):1052–9. doi: 10.1177/1078155219880255 31619132

[39] Al-Azayzih EBAAJZKKAA-K. Inappropriate medications use among elderly cancer patients according to Beer’s criteria. Pharm Pract (Granada). 2024;22(1):1–6.

[40] Paksoy C, Özkan Ö, Ustaalioğlu BB, Sancar M, Demirtunç R, Izzettin FV, et al. Evaluation of potentially inappropriate medication utilization in elderly patients with cancer at outpatient oncology unit. J Oncol Pharm Pract. 2019;25(6):1321–7. doi: 10.1177/1078155218788698 30045684

[41] Wondm SA, Moges TA, Dagnew SB, Dagnew FN, Zeleke TK, Abebe RB, et al. Polypharmacy and potentially inappropriate medicine use in older adults with cancer: A multicenter cross-sectional study in Northwest Ethiopia oncologic centers. J Pharm Policy Pract. 2024;17(1):2397797. doi: 10.1080/20523211.2024.2397797 39359864 PMC11445903

[42] Sajid J, Sabir K, Butt NI, Waris B, Khizar I, Bashir A. Health-Related Quality of Life in Chemotherapy Patients Using the European Organization for Research and Treatment of Cancer Quality of Life Questionnaire-Core 30 (EORTC QLQ-C30): A Single-Institution-Based Study From Lahore, Pakistan. Cureus. 2025;17(6):e86627. doi: 10.7759/cureus.86627 40709163 PMC12287563

[43] Kshirsagar AS, Wani SK. Health-related quality of life in patients with breast cancer surgery and undergoing chemotherapy in Ahmednagar district. J Cancer Res Ther. 2021;17(6):1335–8. doi: 10.4103/jcrt.JCRT_154_19 34916362

[44] Belhaj Haddou M, Igarramen T, Khouchani M, Elkhoudri N. Determinants of health-related quality of life in breast cancer patients: A comprehensive study in Marrakech, Morocco. TOPHJ. 2024;17(1). doi: 10.2174/0118749445317154240729053442

[45] Outlaw D, Dai C, Al-Obaidi M, Harmon C, Giri S, Bhatia S, et al. The association of polypharmacy with functional status impairments, frailty, and health-related quality of life in older adults with gastrointestinal malignancy - Results from the Cancer and Aging Resilience Evaluation (CARE) registry. J Geriatr Oncol. 2022;13(5):624–8. doi: 10.1016/j.jgo.2021.12.010 34998720 PMC9435558

[46] Venturini CD, Engroff P, Ely LS, Zago LF de A, Schroeter G, Gomes I, et al. Gender differences, polypharmacy, and potential pharmacological interactions in the elderly. Clinics (Sao Paulo). 2011;66(11):1867–72. doi: 10.1590/s1807-59322011001100004 22086515 PMC3203957

[47] Kantor ED, Rehm CD, Haas JS, Chan AT, Giovannucci EL. Trends in prescription drug use among adults in the United States from 1999-2012. JAMA. 2015;314(17):1818–31. doi: 10.1001/jama.2015.13766 26529160 PMC4752169

[48] Riechelmann RP, Tannock IF, Wang L, Saad ED, Taback NA, Krzyzanowska MK. Potential drug interactions and duplicate prescriptions among cancer patients. J Natl Cancer Inst. 2007;99(8):592–600. doi: 10.1093/jnci/djk130 17440160

[49] Pud D. Gender differences in predicting quality of life in cancer patients with pain. Eur J Oncol Nurs. 2011;15(5):486–91. doi: 10.1016/j.ejon.2010.12.005 21256079

[50] Laghousi D, Jafari E, Nikbakht H, Nasiri B, Shamshirgaran M, Aminisani N. Gender differences in health-related quality of life among patients with colorectal cancer. J Gastrointest Oncol. 2019;10(3):453–61. doi: 10.21037/jgo.2019.02.04 31183195 PMC6534701

[51] Kim J, Kim Y, Oh JW, Lee S. Sex differences of the association between handgrip strength and health-related quality of life among patients with cancer. Sci Rep. 2024;14(1):9876. doi: 10.1038/s41598-024-60710-6 38684776 PMC11059168

[52] Pyone T, Shamoug A, Chan DZ, Reddy S, Abreu Lopes C. Gender equality for health and well-being: evaluative evidence of interlinkages with other SDGs. New York: UN Women and United Nations University International Institute for Global Health (UNU-IIGH). 2022.

[53] Shrestha S, Sapkota S, Teoh SL, Kc B, Paudyal V, Lee SWH, et al. Clinical pharmacist interventions on pain management in cancer patients (PharmaCAP) in low resource settings: a multicenter feasibility-pilot randomized controlled trial. Support Care Cancer. 2024;32(12):828. doi: 10.1007/s00520-024-08989-z 39601933

[54] Fentie AM, Huluka SA, Gebremariam GT, Gebretekle GB, Abebe E, Fenta TG. Impact of pharmacist-led interventions on medication-related problems among patients treated for cancer: A systematic review and meta-analysis of randomized control trials. Res Social Adm Pharm. 2024;20(5):487–97. doi: 10.1016/j.sapharm.2024.02.006 38368123

