# Supplementary material for: Polypharmacy and quality of life in Yemeni cancer patients undergoing chemotherapy: A cross-sectional study
Source: PLOS Glob Public Health. 2026 Sep 28;6(9):e0006281. doi: 10.1371/journal.pgph.0006281 (PMC13618909; doi:10.1371/journal.pgph.0006281)
Supplement: Supplementary file 1 [file pgph.0006281.s003.pdf]

Thank you for inviting me to review this manuscript which makes a meaningful contribution to the literature on the prevalence and determinants of polypharmacy among cancer patients undergoing chemotherapy in Yemen. The analysis and findings are valuable; however, several methodological clarifications and textual corrections are needed to improve clarity, consistency, and presentation. I hope that my comments below would assist the authors in revising and further strengthening the manuscript.

Methods

Lignes 122–125: This section should be included in the data collection section "*Data were collected through structured face-to-face interviews, and relevant clinical and medication-related information was verified through review of hospital medical records, including details on systemic anti-cancer therapies, supportive medications, and complementary or alternative treatments. Each interview lasted approximately 20–30 minutes*".

Lignes 127–134 : This section should be included in the Study Analysis section : *The study enrolled adult cancer patients receiving chemotherapy to identify predictors of both Global Health Status / Quality of Life (QoL) and polypharmacy using multiple linear regression. Two models were examined: one to assess factors associated with QoL and another to evaluate predictors of polypharmacy, defined as taking >10 non-oncological medications. The independent variables included age group, gender, marital status, occupation, education level, residence, monthly income, and comorbidity status. Participants were recruited at the National Oncology Center in Sana'a during the study period, with eligibility determined by treatment schedule and ethical considerations to minimize burden on patients undergoing active chemotherapy.*

The methodology needs to be better structured in order to avoid redundancy in certain information, to organize the information into the appropriate chapters, and to avoid repetition.

Results

After each frequency (%), include the numerator and denominator (e.g., 46,6% (109/229)).

Conclusion

The conclusion is too long. Please shorten it to emphasize only the key findings and the main message of the study.
