# Supplementary material for: Polypharmacy and quality of life in Yemeni cancer patients undergoing chemotherapy: A cross-sectional study
Source: PLOS Glob Public Health. 2026 Sep 28;6(9):e0006281. doi: 10.1371/journal.pgph.0006281 (PMC13618909; doi:10.1371/journal.pgph.0006281)
Supplement: Supplementary file 2 [file pgph.0006281.s002.docx]

The manuscript addresses an important and still relatively underexplored topic, particularly within a low-resource and conflict-affected setting such as Yemen. It is clear that considerable effort has gone into the work, and overall, the paper is well organized, with a logical progression from the introduction through to the discussion and conclusion. The research question is relevant, and the study adds useful data to a context where evidence remains limited. That said, there are several areas where both the rigor of the analysis and the clarity of presentation could be strengthened to enhance the overall quality and interpretability of the paper.

While the study design itself is appropriate for the stated objectives, some aspects of the interpretation require a more cautious and nuanced approach. In particular, the conclusion that polypharmacy is not associated with quality of life should be tempered. Given that an overwhelming majority of participants were already exposed to polypharmacy, there is very limited variability in the main exposure of interest. This makes it inherently difficult to detect meaningful differences between groups and raises concerns about a potential ceiling effect. As such, the absence of a statistically significant association should not be interpreted too definitively. It would be important for the authors to explicitly acknowledge that this finding may reflect methodological constraints rather than a true absence of effect, and to frame the conclusion accordingly.

The definition of polypharmacy used in the study also warrants further clarification. The decision to focus specifically on non-oncological medications is understandable in the context of supportive care and symptom management; however, this approach differs from a large portion of the existing literature, where polypharmacy is often defined as the total number of medications regardless of indication. Without a clear justification, this choice makes it difficult to compare the reported prevalence with other studies and may lead to some confusion for the reader. Expanding on the rationale behind this definition, and briefly discussing its implications, would help situate the findings more clearly within the broader evidence base.

Another important issue relates to potential confounding. Quality of life in cancer patients is shaped by a complex interplay of clinical, social, and psychological factors. Variables such as cancer stage, treatment intensity, and symptom burden are particularly influential, yet they do not appear to have been included in the analysis. This limits the strength of the conclusions that can be drawn regarding the relationship between polypharmacy and quality of life. It would be important for the authors to acknowledge this more explicitly as a limitation and, where possible, clarify whether these data were unavailable or simply not incorporated into the models. In addition, the relatively modest explanatory power of the regression analyses suggests that key determinants of quality of life may not have been captured, and this should be reflected in the interpretation of the results.

The issue of generalizability also deserves more careful consideration. The study was conducted in a single oncology center, which may not fully reflect the diversity of patient experiences and healthcare access across different regions of Yemen. Given the variability in infrastructure, availability of services, and socioeconomic conditions, it would be helpful to expand the discussion to better contextualize how these findings might—or might not—apply to other settings within the country.

There are also important ethical considerations that could be more thoroughly explored. Although ethical approval and informed consent are reported, the manuscript would benefit from a more reflective discussion of the ethical context in which the study was conducted. In a setting characterized by ongoing conflict and resource constraints, patients may be particularly vulnerable, and participation in research may be influenced by factors such as dependence on healthcare providers or limited access to services. Given that data were collected through face-to-face interviews with patients undergoing chemotherapy, it would be useful to clarify how the research team ensured that participation was fully voluntary, how potential burden was minimized, and how patients were reassured that their care would not be affected by their decision to participate or decline. Addressing these points would strengthen the ethical grounding of the study.

In a similar vein, the role of gender as a significant predictor in the analysis deserves deeper contextualization. The finding that female patients reported lower quality of life and higher levels of polypharmacy is important, but it should not be interpreted in isolation from the broader sociocultural context. In Yemen, gender can reflect underlying disparities in access to care, financial independence, decision-making power, and social support, all of which may influence both health outcomes and medication use. As such, gender may be acting as a proxy for a range of structural and social determinants. A more explicit discussion of these dynamics would help avoid overly simplistic interpretations and provide a more grounded understanding of the findings.

In terms of interpretation more broadly, some sections of the discussion would benefit from a more balanced tone. While the study did not identify a statistically significant association between polypharmacy and quality of life, it is important to recognize that medications in oncology are often prescribed to manage symptoms and improve patient comfort. This means that polypharmacy may have both beneficial and adverse implications, depending on the context. Reflecting this dual perspective would strengthen the discussion and align it more closely with clinical realities.

Finally, the manuscript would benefit from a stronger emphasis on practical implications and recommendations. In particular, the authors may consider drawing on best practices from other low- and middle-income countries facing similar constraints. Examples such as structured medication review programs, integration of clinical pharmacists into oncology care teams, and context-adapted guidelines for deprescribing have shown promise in optimizing medication use and improving patient outcomes. Including a brief discussion of such approaches, and how they might be adapted to the Yemeni context, would enhance the applicability of the findings and provide a more forward-looking perspective.

There are also a few areas where clarity and presentation could be improved. Some of the tables would benefit from clearer labeling, particularly where both percentages and valid percentages are presented, as this may not be immediately intuitive to all readers. The sample size calculation section, while thorough, could be streamlined to improve readability and focus on the key points. In addition, there are minor language inconsistencies and occasional grammatical issues throughout the manuscript that could be addressed through careful editing to enhance overall clarity and flow.

Overall, the study makes a meaningful contribution, particularly given the limited data available from this context. With revisions aimed at strengthening methodological transparency, providing more nuanced interpretation, and improving clarity of presentation, the manuscript would be significantly improved and better positioned for publication.

**Disclosure:**

I confirm that I have no competing interests that could have influenced my assessment of this manuscript. I do not have any financial, institutional, or personal relationships with the authors or the work presented that would affect my ability to provide an objective and unbiased review.

I also confirm that I am not aware of any similar or overlapping manuscript authored by me or under consideration elsewhere that would constitute duplication or conflict with this submission.

I have conducted this review in accordance with PLOS ethical peer review standards and COPE guidelines, ensuring confidentiality of the manuscript and its contents. I have not shared or used any part of the submission outside the scope of this review, and I have not uploaded or processed the manuscript content in any external platforms or tools.

Any minor use of AI-assisted tools was limited strictly to language editing and proofreading of my review comments, and no manuscript content was uploaded or used in such tools. All evaluations, interpretations, and recommendations expressed in this review are entirely my own.
AI-assisted tools were used solely for proofreading and minor grammar refinement of this review. All substantive comments, interpretations, and recommendations reflect my independent assessment.
