# Supplementary material for: Polypharmacy and quality of life in Yemeni cancer patients undergoing chemotherapy: A cross-sectional study
Source: PLOS Glob Public Health. 2026 Sep 28;6(9):e0006281. doi: 10.1371/journal.pgph.0006281 (PMC13618909; doi:10.1371/journal.pgph.0006281)
Supplement: Supplementary file 3 [file pgph.0006281.s004.docx]

Reviewer’s comments **PGPH-D-26-01135**

1. **Comment 1 — Objective misalignment**
   The study’s stated objective (lines 107–109) indicate that the study aimed to examine “the prevalence and determinants of polypharmacy among cancer patients undergoing chemotherapy.”

However, the analyses and results actually concern only non‑oncological medications: chemotherapy and other systemic anti‑cancer agents were explicitly excluded from the polypharmacy count.

Please revise/ rephrase the objective to accurately reflect that polypharmacy was assessed for non‑oncological (supportive and concomitant) medications only.

Please also ensure consistency in the objective, methods, results and discussion.

2. **Comment 2 — Sample size justification**
   The sample size justification (Section 2.2, lines 134–139) currently relies solely on Green’s rule of thumb (50 + 8m). That is a useful starting point but does not replace a formal a priori power analysis for studies that use multiple regression. Please provide a formal power calculation for each regression model, explicitly reporting: the expected effect size (f² or R²) based on prior studies or a pilot estimate, the desired power (conventionally ≥ 0.80), the alpha level, and the number of predictors in each model. It is suggested to cite Green’s rule as secondary confirmation, but if a prospective power analysis was not performed, state this clearly as a limitation.
3. **Comment 3 — Definition of polypharmacy**
   There is a factual inconsistency and a scientific error in definition of polypharmacy. On page 3 (lines 81–86) the authors refer to the WHO definition (concurrent use of five or more medications, ≥5). However, on page 6 (lines 151–152) the manuscript states that polypharmacy was defined as “more than five non‑oncological medications,” which implies counting only non‑oncological drugs and sets the threshold at >5 (i.e., six or more). This statement is incorrect in two ways: (1) it mis-states the standard threshold by changing ≥5 to >5, and (2) it limits the definition to non‑oncological medications without making that restriction explicit and justified. Please correct and harmonise the definition across the manuscript. If intent was to count only non – oncology (supportive medication), explicitly state this in the objective, methods and justify why oncological agents were excluded because changing the denominator alter prevalence estimates and comparability with existing literature. Also provide citation supporting the chosen numerical threshold.
4. **Comment 4 — Missing clinical variables**
   Table 1 lists sociodemographic and clinical characteristics but omits several clinically important variables that are likely to affect medication use and quality of life. In particular, please add: cancer stage (for example early vs advanced/metastatic), and detailed categories of comorbidities (for example diabetes, hypertension, cardiovascular disease) rather than simply presence/absence. Where feasible, include these variables as covariates in the regression models because they may confound the relationship between polypharmacy and quality of life.
5. **Comment 5 — SOS medication reporting**
   In Table 2, Paracetamol and Furosemide are labelled “(SOS)” while other medications have no schedule information. This selective labelling creates ambiguity about whether the other medicines were scheduled or also taken as needed. Please make the notation consistent for all medications, or add a footnote explaining how SOS medications were recorded and whether they were counted toward the polypharmacy threshold. Note that including intermittently used (SOS) drugs can inflate prevalence estimates, so provide a methodological justification if they were counted.
6. **Comment 6 — Classification of natural products**
   Table 2 lists honey, garlic, ginger, black seed, and caper bush under “Complementary/Alternative Medications.” These items are food/herbal products rather than conventional pharmacologic medicines, so classifying them as “medications” and including them in the medication count is a matter of debate. Please reclassify them under a separate heading (for example “Natural products/ Herbal remedies/ Traditional/Complementary products”) and clarify whether they were included in the polypharmacy count. Provide a clear rationale for the decision, since including such items may overestimate pharmacological burden.
7. **Comment 7 — Unhelpful columns in Table 3**
   Table 3 includes N, Minimum and Maximum columns that add no useful information: N is identical across rows (n = 234) and Min/Max are uniformly 0 and 100, which only reflect the theoretical scoring range of the EORTC QLQ‑C30. Replace these with more informative distributional statistics such as the median and interquartile range (IQR) and/or 95% confidence intervals for the mean. Given the large SDs and likely skewness or floor/ceiling effects for some scales, median and IQR would be especially informative and align with best practice for HRQoL data.
8. **Comment 8 — Analytical sample for regression (Table 5)**
   The heading of Table 5 reads “Multiple Linear Regression Analysis of Factors Associated with the Number of Non‑oncological Medications (n = 132),” which suggests the model was run only among the 132 participants with excessive polypharmacy (>10). If so, this is a major problem: predictors of medication count should be modelled in the full sample (n = 234). Restricting the analysis to those already above a threshold introduces selection bias and limits generalisability. Please clarify which analytical sample was used; if the regression was restricted to n = 132, re‑run it on the complete sample and update the table and model statistics (F, R², degrees of freedom) so they are internally consistent.
