# Supplementary material for: Polypharmacy and quality of life in Yemeni cancer patients undergoing chemotherapy: A cross-sectional study
Source: PLOS Glob Public Health. 2026 Sep 28;6(9):e0006281. doi: 10.1371/journal.pgph.0006281 (PMC13618909; doi:10.1371/journal.pgph.0006281)
Supplement: Supplementary file 4 [file pgph.0006281.s005.docx]

28-06-2026

Dear Dr. Catherine Kyobutungi

Editor-in-Chief
PLOS Global Public Health

Thank you for your decision letter dated 21 May 2026 and for giving us the opportunity to submit a revised version of our manuscript, “**Polypharmacy and Quality of Life in Yemeni Cancer Patients Undergoing Chemotherapy: A Cross-Sectional Study**”

We are sincerely grateful to the reviewers for their thoughtful and valuable comments. We carefully considered each point raised and have revised the manuscript accordingly. We believe these changes have further strengthened the clarity and rigor of the work. As the first study of its kind in Yemen, it offers valuable insights that will enrich the broader scientific understanding of this topic.

Enclosed is our detailed point-by-point response to the editor’s and reviewers’ comments. For ease of review, major revisions in the manuscript have been highlighted in Yellow.

We truly appreciate your positive decision and the opportunity to resubmit. We hope the revised manuscript will now meet the expectations for publication in PLOS Global Public Health.

We look forward to hearing from you.

Yours Sincerely,

Sayida Al-Jamei (PhD)

Department of Clinical Pharmacy. School of Medical Science and Pharmacy, Lebanese International University, Sana’a, Yemen

Email: [saydaahmed@yahoo.com](mailto:saydaahmed@yahoo.com)

The response:

Thank you so much for this decision. We provided below one by one responses to the reviewer’s comments (blue color)

Journal Requirements:

1. Please ensure that your Ethics Statement is available in its entirety at the beginning of your Methods section, under a subheading 'Ethics Statement'.

Thank you for this comment. We have revised the manuscript accordingly. A complete Ethics Statement has now been included at the beginning of the Methods section. The statement specifies the ethics committee approval number and confirms that informed consent was obtained from all participants prior to their participation in the study.

2. Tables should not be uploaded as individual files. Please remove these files and include the Tables in your manuscript file as editable, cell-based objects. For more information about how to format tables, see our guidelines: [LINK]

https://journals.plos.org/globalpublichealth/s/tables

Thank you for bringing this to our attention. We have removed the separately uploaded table files and incorporated all tables directly into the manuscript as editable, cell-based tables in accordance with the journal’s formatting requirements.

3. We note that your Data Availability Statement is currently as follows: "The authors confirm that the data supporting the findings of this study are available within the article"

 Please confirm at this time whether or not your submission contains all raw data required to replicate the results of your study. Authors must share the “minimal data set” for their submission. PLOS defines the minimal data set to consist of the data required to replicate all study findings reported in the article, as well as related metadata and methods

(https://journals.plos.org/plosone/s/data-availability#loc-minimal-data-set-definition).
 
 For example, authors should submit the following data:

 - The values behind the means, standard deviations and other measures reported;

 - The values used to build graphs;

 - The points extracted from images for analysis.

Authors do not need to submit their entire data set if only a portion of the data was used in the reported study.

If your submission does not contain these data, please either upload them as Supporting Information files or deposit them to a stable, public repository and provide us with the relevant URLs, DOIs, or accession numbers. For a list of recommended repositories, please see https://journals.plos.org/plosone/s/recommended-repositories.
 
Thank you for highlighting this requirement. We confirm that the submission now includes the minimal data set necessary to replicate the findings reported in the manuscript. The data contain the variables underlying the analyses presented in the study, including the values used to generate summary statistics and reported results. The dataset has been provided as Supporting Information and the Data Availability Statement has been updated accordingly.

**Reviewer comments**

Reviewer #1: Thank you for the opportunity to review this manuscript. The study addresses an important and underexplored topic, particularly within a resource-limited and conflict-affected setting such as Yemen, and contributes valuable preliminary data on polypharmacy and quality of life among cancer patients undergoing chemotherapy.

Overall, the manuscript is well structured and clearly written, with a logical flow from introduction to conclusion. However, several areas require further clarification and strengthening. The interpretation of the findings, particularly the lack of association between polypharmacy and quality of life, should be presented more cautiously given the limited variability in exposure, as the vast majority of participants were already experiencing polypharmacy. This may have limited the ability to detect meaningful differences.

We thank the reviewer for the positive evaluation of our manuscript and for this valuable comment. We agree that the high prevalence of polypharmacy in our cohort may have limited the variability in exposure and consequently reduced our ability to detect an independent association between polypharmacy and quality of life. In response, we have revised the Discussion section to present this finding more cautiously and have explicitly acknowledged that the lack of a significant association should be interpreted in light of the limited exposure variability and the cross-sectional design. This limitation has also been emphasized in the Limitations section.

In addition, further justification is needed for the definition of polypharmacy focusing exclusively on non-oncological medications, as this differs from commonly used definitions in the literature.

We thank the reviewer for this important comment. We acknowledge that definitions of polypharmacy vary across the literature, and many studies include all prescribed medications regardless of indication. However, in the present study, we intentionally focused on non-oncological medications and excluded systemic anti-cancer agents to more accurately capture the modifiable medication burden related to comorbid conditions and supportive care needs, rather than protocol-driven chemotherapy regimens.

This approach is particularly relevant in oncology settings, where anticancer therapies are standardized and disease-specific, and their inclusion may overestimate medication burden without reflecting potentially avoidable or modifiable prescribing practices. Therefore, our definition aims to better reflect clinically meaningful concomitant medication load in cancer patients.

We have now clarified and strengthened this rationale in the Methods section to improve transparency and alignment with the study objectives.

There are also important considerations related to potential confounding that should be more explicitly acknowledged. Key clinical variables such as cancer stage, treatment intensity, and symptom severity were not included in the analysis, although they are likely to have a significant impact on quality of life outcomes. The relatively low explanatory power of the regression models further suggests that additional relevant factors may not have been captured.

We thank the reviewer for this important observation. We agree that clinical variables such as cancer stage, treatment intensity, and symptom severity are important determinants of quality of life in cancer patients and may act as potential confounders.

Cancer stage was included in the descriptive analysis (Table 1) to better characterize the study population. However, due to incomplete and inconsistently documented clinical records, it was not possible to include cancer stage in the multivariable regression models. Similarly, detailed information on treatment intensity and symptom severity was not consistently available in the dataset, reflecting limitations in routine clinical documentation in the study setting.

We acknowledge that the absence of these variables may have contributed to the relatively modest explanatory power of the regression models. This limitation has been explicitly addressed in the revised manuscript. Nevertheless, the study provides valuable preliminary evidence on sociodemographic, clinical, and medication-related predictors of quality of life in a resource-limited setting where such data remain scarce.

The manuscript would also benefit from a more in-depth discussion of contextual factors, particularly the role of gender. The finding that female patients experience lower quality of life and higher levels of polypharmacy should be interpreted within the broader sociocultural and structural context of Yemen, where gender-related disparities in healthcare access, financial autonomy, and social support may act as important confounders.

We thank the reviewer for this insightful comment. We agree that the observed gender differences should be interpreted within the broader sociocultural and structural context of Yemen. In response, we have expanded the Discussion to more explicitly address how gender-related disparities in healthcare access, financial autonomy, and social support may influence both medication burden and quality of life outcomes.

In the revised manuscript, we emphasize that women in resource-limited settings such as Yemen may face additional barriers in accessing timely care, affording medications, and making independent healthcare decisions, which may contribute to poorer quality of life and differences in medication use patterns. We also acknowledge that these structural factors may act as unmeasured confounders influencing the observed associations.

These revisions strengthen the contextual interpretation of our findings and provide a more comprehensive explanation of the gender-related differences observed in this study.

In addition, ethical considerations could be more comprehensively addressed. While ethical approval and consent procedures are mentioned, further reflection on participant vulnerability, the clinical setting of recruitment, and measures taken to ensure voluntary participation would strengthen the manuscript.

We thank the reviewer for this valuable suggestion. In response, we have expanded the Ethics section to further address participant vulnerability and recruitment procedures. We clarify that cancer patients undergoing chemotherapy represent a vulnerable population and that recruitment was conducted in a clinical setting with careful attention to avoiding any perceived coercion. Participants were clearly informed that their decision to participate or decline would not affect their treatment, follow-up care, or access to services, thereby ensuring voluntary participation. These additions have been incorporated into the revised manuscript to strengthen the ethical considerations.

Finally, some minor revisions related to clarity, consistency in terminology, and table presentation are recommended to improve readability.
Please see the attached document for more detailed comments and specific suggestions for revision.

We thank the reviewer for the constructive feedback and for the careful review of our manuscript. We appreciate the suggestion regarding minor revisions related to clarity, consistency in terminology, and table presentation. In response, we have carefully reviewed the entire manuscript and revised the text to improve clarity, ensure consistency in terminology, and enhance the presentation of tables for better readability. All specific comments provided in the annotated document have been addressed accordingly in the revised version.

**Reviewer comments**

The manuscript addresses an important and still relatively underexplored topic, particularly within a low-resource and conflict-affected setting such as Yemen. It is clear that considerable effort has gone into the work, and overall, the paper is well organized, with a logical progression from the introduction through to the discussion and conclusion. The research question is relevant, and the study adds useful data to a context where evidence remains limited. That said, there are several areas where both the rigor of the analysis and the clarity of presentation could be strengthened to enhance the overall quality and interpretability of the paper.

We sincerely thank the reviewer for the positive evaluation of our manuscript and for recognizing the importance and relevance of this research in the context of Yemen. We appreciate the constructive suggestions provided. In response, we have carefully revised the manuscript to strengthen the rigor of the analyses, improve the clarity of presentation, and enhance the overall organization and interpretability of the findings. Specific revisions addressing each of the reviewer's comments are detailed in the responses below.

While the study design itself is appropriate for the stated objectives, some aspects of the interpretation require a more cautious and nuanced approach. In particular, the conclusion that polypharmacy is not associated with quality of life should be tempered. Given that an overwhelming majority of participants were already exposed to polypharmacy, there is very limited variability in the main exposure of interest. This makes it inherently difficult to detect meaningful differences between groups and raises concerns about a potential ceiling effect. As such, the absence of a statistically significant association should not be interpreted too definitively. It would be important for the authors to explicitly acknowledge that this finding may reflect methodological constraints rather than a true absence of effect, and to frame the conclusion accordingly.

We thank the reviewer for this important and thoughtful comment. We agree that the high prevalence of polypharmacy in our study population resulted in limited variability in the exposure, making it more difficult to detect meaningful differences in quality of life. In response, we have revised the Methods, Discussion, Limitations, and Conclusion sections to clarify that, because nearly all participants met the conventional definition of polypharmacy (≥5 non-oncological medications), subsequent analyses focused on excessive polypharmacy (>10 medications) to improve statistical discrimination. We have also tempered our interpretation of the findings by explicitly stating that the lack of a statistically significant association should not be interpreted as evidence that polypharmacy has no effect on quality of life. Instead, we acknowledge that the limited variability in medication exposure and the resulting reduced statistical contrast may have limited our ability to detect modest associations. We believe these revisions provide a more balanced and appropriately cautious interpretation of the findings.

The definition of polypharmacy used in the study also warrants further clarification. The decision to focus specifically on non-oncological medications is understandable in the context of supportive care and symptom management; however, this approach differs from a large portion of the existing literature, where polypharmacy is often defined as the total number of medications regardless of indication. Without a clear justification, this choice makes it difficult to compare the reported prevalence with other studies and may lead to some confusion for the reader. Expanding on the rationale behind this definition, and briefly discussing its implications, would help situate the findings more clearly within the broader evidence base.

We thank the reviewer for this important comment. We agree that definitions of polypharmacy vary across the literature, particularly regarding whether all medications or only non-oncological medications are included. In our study, we defined polypharmacy based on non-oncological medications only, while excluding systemic anti-cancer agents, in order to more specifically capture the medication burden related to comorbid conditions and supportive care rather than protocol-driven chemotherapy regimens, which are typically not modifiable in routine clinical practice. We have now clarified this rationale more explicitly in the Methods section (Study Instruments) and highlighted in the Discussion that this definition may limit direct comparability with studies using total medication counts. However, we believe this approach provides a more clinically meaningful assessment of potentially avoidable medication burden in this population.

Another important issue relates to potential confounding. Quality of life in cancer patients is shaped by a complex interplay of clinical, social, and psychological factors. Variables such as cancer stage, treatment intensity, and symptom burden are particularly influential, yet they do not appear to have been included in the analysis. This limits the strength of the conclusions that can be drawn regarding the relationship between polypharmacy and quality of life. It would be important for the authors to acknowledge this more explicitly as a limitation and, where possible, clarify whether these data were unavailable or simply not incorporated into the models. In addition, the relatively modest explanatory power of the regression analyses suggests that key determinants of quality of life may not have been captured, and this should be reflected in the interpretation of the results.

We thank the reviewer for this important observation. We agree that clinical variables such as cancer stage, treatment intensity, and symptom severity are important determinants of quality of life in cancer patients and may act as potential confounders.

Cancer stage was included in the descriptive analysis (Table 1) to better characterize the study population. However, due to incomplete and inconsistently documented clinical records, it was not possible to include cancer stage in the multivariable regression models. Similarly, detailed information on treatment intensity and symptom severity was not consistently available in the dataset, reflecting limitations in routine clinical documentation in the study setting.

We acknowledge that the absence of these variables may have contributed to the relatively modest explanatory power of the regression models. This limitation has been explicitly addressed in the revised manuscript. Nevertheless, the study provides valuable preliminary evidence on sociodemographic, clinical, and medication-related predictors of quality of life in a resource-limited setting where such data remain scarce.

The issue of generalizability also deserves more careful consideration. The study was conducted in a single oncology center, which may not fully reflect the diversity of patient experiences and healthcare access across different regions of Yemen. Given the variability in infrastructure, availability of services, and socioeconomic conditions, it would be helpful to expand the discussion to better contextualize how these findings might—or might not—apply to other settings within the country.

We thank the reviewer for this important observation. We agree that the single-center design may limit the generalizability of our findings to other regions of Yemen, given the heterogeneity in healthcare infrastructure, access to oncology services, and socioeconomic conditions across the country. In response, we have expanded the Discussion and Limitations sections to more clearly acknowledge this issue and to contextualize our findings within the broader Yemeni healthcare setting. We now emphasize that our study was conducted in a major tertiary referral center, which may not fully represent patients treated in peripheral or resource-limited facilities, and that caution is therefore needed when extrapolating these results to the national level.

There are also important ethical considerations that could be more thoroughly explored. Although ethical approval and informed consent are reported, the manuscript would benefit from a more reflective discussion of the ethical context in which the study was conducted. In a setting characterized by ongoing conflict and resource constraints, patients may be particularly vulnerable, and participation in research may be influenced by factors such as dependence on healthcare providers or limited access to services. Given that data were collected through face-to-face interviews with patients undergoing chemotherapy, it would be useful to clarify how the research team ensured that participation was fully voluntary, how potential burden was minimized, and how patients were reassured that their care would not be affected by their decision to participate or decline. Addressing these points would strengthen the ethical grounding of the study.

We thank the reviewer for this valuable and important comment. We agree that the ethical context of conducting research among cancer patients in a resource-limited and conflict-affected setting warrants further clarification. In response, we have expanded the Ethics Approval and Informed Consent section to provide greater detail on how participant vulnerability was addressed. Specifically, we clarified that participation was strictly voluntary, that patients were informed that refusal to participate would not affect their clinical care in any way, and that informed consent was obtained prior to any data collection. We also emphasize that interviews were conducted in a private and respectful manner, with efforts made to minimize patient burden by keeping interviews concise and aligning data collection with routine clinical visits. These clarifications have been added to strengthen the transparency and ethical rigor of the study.

In a similar vein, the role of gender as a significant predictor in the analysis deserves deeper contextualization. The finding that female patients reported lower quality of life and higher levels of polypharmacy is important, but it should not be interpreted in isolation from the broader sociocultural context. In Yemen, gender can reflect underlying disparities in access to care, financial independence, decision-making power, and social support, all of which may influence both health outcomes and medication use. As such, gender may be acting as a proxy for a range of structural and social determinants. A more explicit discussion of these dynamics would help avoid overly simplistic interpretations and provide a more grounded understanding of the findings.

We thank the reviewer for this insightful comment. We agree that the observed gender differences should be interpreted within the broader sociocultural and structural context of Yemen. In response, we have expanded the Discussion to more explicitly address how gender-related disparities in healthcare access, financial autonomy, and social support may influence both medication burden and quality of life outcomes.

In the revised manuscript, we emphasize that women in resource-limited settings such as Yemen may face additional barriers in accessing timely care, affording medications, and making independent healthcare decisions, which may contribute to poorer quality of life and differences in medication use patterns. We also acknowledge that these structural factors may act as unmeasured confounders influencing the observed associations.

These revisions strengthen the contextual interpretation of our findings and provide a more comprehensive explanation of the gender-related differences observed in this study.

In terms of interpretation more broadly, some sections of the discussion would benefit from a more balanced tone. While the study did not identify a statistically significant association between polypharmacy and quality of life, it is important to recognize that medications in oncology are often prescribed to manage symptoms and improve patient comfort. This means that polypharmacy may have both beneficial and adverse implications, depending on the context. Reflecting this dual perspective would strengthen the discussion and align it more closely with clinical realities.

We thank the reviewer for this insightful comment. We agree that polypharmacy in oncology is complex and may have both beneficial and potentially harmful effects depending on the clinical context. In response, we have revised the Discussion section to adopt a more balanced interpretation by explicitly acknowledging that many concomitant medications are prescribed for symptom control and supportive care (e.g., antiemetics, analgesics, and corticosteroids), which may improve patient comfort and quality of life. We also emphasize that, alongside these benefits, polypharmacy may still carry risks related to drug–drug interactions and treatment complexity. These revisions aim to better reflect the dual nature of medication burden in oncology and align the interpretation with clinical practice.

There are also a few areas where clarity and presentation could be improved. Some of the tables would benefit from clearer labeling, particularly where both percentages and valid percentages are presented, as this may not be immediately intuitive to all readers. The sample size calculation section, while thorough, could be streamlined to improve readability and focus on the key points. In addition, there are minor language inconsistencies and occasional grammatical issues throughout the manuscript that could be addressed through careful editing to enhance overall clarity and flow.

We thank the reviewer for these helpful suggestions. In response, we have carefully revised the manuscript to improve clarity and presentation. The tables have been updated with clearer labeling, and we have simplified the presentation of percentages by ensuring consistent reporting and clearer interpretation where applicable. The sample size calculation section has been streamlined to improve readability while retaining all essential methodological details. In addition, the manuscript has undergone thorough language editing to correct grammatical issues and improve overall clarity, consistency, and flow throughout the text.

Overall, the study makes a meaningful contribution, particularly given the limited data available from this context. With revisions aimed at strengthening methodological transparency, providing more nuanced interpretation, and improving clarity of presentation, the manuscript would be significantly improved and better positioned for publication.

We thank the reviewer for this positive overall assessment and for the constructive feedback provided throughout the review. We appreciate the recognition of the relevance and contribution of our study, particularly in a resource-limited and under-researched setting. We have carefully revised the manuscript in line with all comments, with particular attention to improving methodological clarity, strengthening the interpretation of findings, and enhancing the overall clarity and presentation of the text. We believe these revisions have significantly improved the quality of the manuscript and its suitability for publication.

Reviewer #2: This is an original recent work. No searched articles are similar or replicates of this study. The study was carefully planned, executed and recorded. The conclusions align well with the findings and the objectives of the study. Appropriate ethical procedures were taken and reported. The whole presentation is logical and intelligible. Data availability to the public has been declared.

We thank the reviewer for this positive evaluation of our manuscript. We sincerely appreciate the recognition of the originality of the work, the methodological rigor, and the clear alignment between the study objectives, findings, and conclusions. We are also grateful for the acknowledgement of the ethical standards followed and the overall clarity of the presentation. Your encouraging feedback is highly valued and strengthens the significance of our study.

Reviewer #3: Thank you for inviting me to review this manuscript which makes a meaningful contribution to the literature on the prevalence and determinants of polypharmacy among cancer patients undergoing chemotherapy in Yemen. The analysis and findings are valuable; however, several methodological clarifications and textual corrections are needed to improve clarity, consistency, and presentation. I hope that my comments below would assist the authors in revising and further strengthening the manuscript.

We thank the reviewer for taking the time to review our manuscript and for the constructive and insightful comments provided. We appreciate the recognition of the relevance and contribution of our study to the limited evidence on polypharmacy among cancer patients in Yemen. We have carefully addressed all methodological clarifications and textual suggestions, and revised the manuscript accordingly to improve clarity, consistency, and overall presentation.

**Reviewer comments**

Reviewer #3: Thank you for inviting me to review this manuscript which makes a meaningful contribution to the literature on the prevalence and determinants of polypharmacy among cancer patients undergoing chemotherapy in Yemen. The analysis and findings are valuable; however, several methodological clarifications and textual corrections are needed to improve clarity, consistency, and presentation. I hope that my comments below would assist the authors in revising and further strengthening the manuscript.

We thank the reviewer for the positive evaluation of our manuscript and for recognizing its contribution to the literature on polypharmacy among cancer patients in Yemen. We also appreciate the constructive comments and suggestions, which have been very helpful in improving the clarity, consistency, and overall quality of the manuscript. We have carefully revised the manuscript in response to all comments, and we believe these revisions have strengthened the methodological clarity and presentation of the study.

Lines 122–125: This section should be included in the data collection section “Data were collected through structured face-to-face interviews, and relevant clinical and medication-related information was verified through review of hospital medical records, including details on systemic anti-cancer therapies, supportive medications, and complementary or alternative treatments. Each interview lasted approximately 20–30 minutes”.

We thank the reviewer for this comment. We confirm that data collection procedures have been clearly described in the Methods section. Specifically, we have stated that data were collected through structured face-to-face interviews, and that relevant clinical and medication-related information was verified through hospital medical records, including systemic anti-cancer therapies, supportive medications, and complementary or alternative treatments. We have also specified the approximate duration of each interview (20–30 minutes). No further changes were required, as this information is already clearly included in the revised manuscript.

Lines 127–134 : This section should be included in the Study Analysis section : The study enrolled adult cancer patients receiving chemotherapy to identify predictors of both Global Health Status / Quality of Life (QoL) and polypharmacy using multiple linear regression. Two models were examined: one to assess factors associated with QoL and another to evaluate predictors of polypharmacy, defined as taking >10 non-oncological medications. The independent variables included age group, gender, marital status, occupation, education level, residence, monthly income, and comorbidity status. Participants were recruited at the National Oncology Center in Sana’a during the study period, with eligibility determined by treatment schedule and ethical considerations to minimize burden on patients undergoing active chemotherapy.

We thank the reviewer for this helpful suggestion. In response, we have revised the Methods section to improve structural clarity and consistency. The content describing the analytical approach, including the study objectives, regression models, and the variables included in the analysis, has now been moved and incorporated into the Study Analysis section. This revision ensures that all statistical analysis procedures are presented in a single, clearly defined section, improving readability and methodological coherence.

The methodology needs to be better structured in order to avoid redundancy in certain information, to organize the information into the appropriate chapters, and to avoid repetition.

We thank the reviewer for this valuable comment. The Methods section has been thoroughly revised to improve structure, clarity, and flow. Redundant statements have been removed, and overlapping content has been reorganized into the most appropriate subsections (Study Design and Participants, Sample Size Calculation, Study Instruments, and Study Analysis). This revision reduces repetition and ensures that each methodological component is clearly and consistently presented in its correct section, improving overall readability and coherence of the manuscript.

Results

After each frequency (%), include the numerator and denominator (e.g., 46,6% (109/229)).

We thank the reviewer for this helpful suggestion. The Results section has been revised accordingly, and all percentages are now consistently reported together with their corresponding numerator and denominator (n/N). This improvement enhances transparency and allows clearer interpretation of the reported frequencies throughout the manuscript.

Conclusion

The conclusion is too long. Please shorten it to emphasize only the key findings and the main message of the study.

We thank the reviewer for this helpful suggestion. The Conclusion has been substantially shortened and revised to emphasize the principal findings and the main clinical message of the study, while removing repetitive details presented in the Discussion. This revision improves the clarity, focus, and overall readability of the manuscript.

Reviewer #4: It is a relevant piece of work. The findings shall be of use to the prescribers and very likely improve the safer use of medications.
It is proposed to address the comments in order to be scientifically sound.

We thank the reviewer for this positive appraisal of our work and for recognizing its potential relevance to clinical practice and medication safety. We appreciate the constructive recommendation and have carefully addressed all comments to further strengthen the scientific rigor, clarity, and overall quality of the manuscript.

**Reviewer comments**

**Comment 1 — Objective misalignment**

The study’s stated objective (lines 107–109) indicate that the study aimed to examine “the prevalence and determinants of polypharmacy among cancer patients undergoing chemotherapy.”

However, the analyses and results actually concern only non‑oncological medications: chemotherapy and other systemic anti‑cancer agents were explicitly excluded from the polypharmacy count.

Please revise/ rephrase the objective to accurately reflect that polypharmacy was assessed for non‑oncological (supportive and concomitant) medications only.

Please also ensure consistency in the objective, methods, results and discussion.

Thank you for this valuable observation. We agree that the original wording of the objective could imply that all medications, including anticancer therapies, were considered in the assessment of polypharmacy. To improve clarity and ensure consistency throughout the manuscript, we have revised the objective to explicitly state that polypharmacy was assessed based on non-oncological medications (supportive and concomitant medications), excluding chemotherapy and other systemic anticancer agents. Corresponding revisions have also been made in the Methods, Results, and Discussion sections to maintain consistency.

**Comment 2 — Sample size justification**

The sample size justification (Section 2.2, lines 134–139) currently relies solely on Green’s rule of thumb (50 + 8m). That is a useful starting point but does not replace a formal a priori power analysis for studies that use multiple regression. Please provide a formal power calculation for each regression model, explicitly reporting: the expected effect size (f² or R²) based on prior studies or a pilot estimate, the desired power (conventionally ≥ 0.80), the alpha level, and the number of predictors in each model. It is suggested to cite Green’s rule as secondary confirmation, but if a prospective power analysis was not performed, state this clearly as a limitation.

We thank the reviewer for this important suggestion. We have revised the sample size justification section to include a formal post-hoc power estimation based on observed effect sizes (f² derived from R² values). The wording has been carefully adjusted to present power as an estimated value rather than an absolute calculated result, in line with statistical reporting standards for observational regression analyses. Green’s rule of thumb was retained as a supplementary justification, and the absence of a priori power analysis has been acknowledged as a limitation.

**Comment 3 — Definition of polypharmacy**

There is a factual inconsistency and a scientific error in definition of polypharmacy. On page 3 (lines 81–86) the authors refer to the WHO definition (concurrent use of five or more medications, ≥5). However, on page 6 (lines 151–152) the manuscript states that polypharmacy was defined as “more than five non‑oncological medications,” which implies counting only non‑oncological drugs and sets the threshold at >5 (i.e., six or more). This statement is incorrect in two ways: (1) it mis-states the standard threshold by changing ≥5 to >5, and (2) it limits the definition to non‑oncological medications without making that restriction explicit and justified. Please correct and harmonise the definition across the manuscript. If intent was to count only non – oncology (supportive medication), explicitly state this in the objective, methods and justify why oncological agents were excluded because changing the denominator alter prevalence estimates and comparability with existing literature. Also provide citation supporting the chosen numerical threshold.

We thank the reviewer for this important observation. We acknowledge that the definition of polypharmacy was inconsistently described across the manuscript.

In response, we have revised and harmonized the definition throughout the manuscript to ensure consistency with the WHO definition and standard clinical terminology. Specifically, we clarified that polypharmacy refers to the concurrent use of five or more medications (≥5), while excessive polypharmacy refers to the use of more than 10 medications. We also clarified that the present study focused on non-oncological (supportive and concomitant) medications, excluding systemic anti-cancer therapies, and justified this approach due to the need to assess medication burden beyond chemotherapy-related treatment regimens.

All relevant sections (Introduction, Methods, Study Instruments, and Study Analysis) have been revised accordingly, and appropriate clarification has been added.

**Comment 4 — Missing clinical variables**

Table 1 lists sociodemographic and clinical characteristics but omits several clinically important variables that are likely to affect medication use and quality of life. In particular, please add: cancer stage (for example early vs advanced/metastatic), and detailed categories of comorbidities (for example diabetes, hypertension, cardiovascular disease) rather than simply presence/absence. Where feasible, include these variables as covariates in the regression models because they may confound the relationship between polypharmacy and quality of life.

We thank the reviewer for this valuable suggestion. In response, we have revised Table 1 to include additional clinically relevant variables, specifically cancer stage to better characterize the study population.

Cancer stage (early vs advanced) has now been added to Table 1. However, it should be noted that cancer stage data were not consistently documented in the medical records for all patients, reflecting variability in oncology record-keeping in the study setting. As a result, a proportion of cases had missing staging information.

Importantly, due to the extent of missing and incomplete clinical documentation, cancer stage was not included in the regression analyses to avoid introducing bias and misclassification in the adjusted models.

**Comment 5 — SOS medication reporting**

In Table 2, Paracetamol and Furosemide are labelled “(SOS)” while other medications have no schedule information. This selective labelling creates ambiguity about whether the other medicines were scheduled or also taken as needed. Please make the notation consistent for all medications, or add a footnote explaining how SOS medications were recorded and whether they were counted toward the polypharmacy threshold. Note that including intermittently used (SOS) drugs can inflate prevalence estimates, so provide a methodological justification if they were counted.

We thank the reviewer for this important comment. We agree that the initial presentation of SOS (as-needed) medications in Table 2 could create ambiguity regarding medication scheduling.

In response, we have added a clarifying footnote to Table 2 explaining how SOS medications were defined and recorded. Specifically, paracetamol and furosemide were documented as SOS medications according to prescribing records, while other medications were recorded as routinely scheduled therapies.

The footnote further clarifies that SOS medications were included in the total medication count when documented in the medical records, regardless of frequency of actual use, and were therefore considered in the assessment of polypharmacy. This approach is consistent with previously published studies that define polypharmacy based on prescribed medications rather than actual daily intake.

**Comment 6 — Classification of natural products**

Table 2 lists honey, garlic, ginger, black seed, and caper bush under “Complementary/Alternative Medications.” These items are food/herbal products rather than conventional pharmacologic medicines, so classifying them as “medications” and including them in the medication count is a matter of debate. Please reclassify them under a separate heading (for example “Natural products/ Herbal remedies/ Traditional/Complementary products”) and clarify whether they were included in the polypharmacy count. Provide a clear rationale for the decision, since including such items may overestimate pharmacological burden.

We thank the reviewer for this important observation. We agree that honey, garlic, ginger, black seed, and caper bush are more appropriately classified as natural products or traditional remedies rather than conventional medications.

Accordingly, Table 2 has been revised to present these items under a separate heading, “Natural Products / Herbal and Traditional Remedies,” to clearly distinguish them from pharmacological therapies. We have also added clarifying text in the Methods section and a footnote to Table 2 regarding their classification and treatment in the analysis.

These products were included in the medication count because the objective of the study was to assess the overall medication burden among cancer patients, including self-administered products used for therapeutic purposes alongside conventional treatment. Given the widespread use of traditional and herbal remedies in the study population, excluding these products may have underestimated patients’ total treatment burden and concurrent product use. To ensure transparency, the revised manuscript explicitly states that natural and herbal products were included in the polypharmacy assessment

**Comment 7 — Unhelpful columns in Table 3**

Table 3 includes N, Minimum and Maximum columns that add no useful information: N is identical across rows (n = 234) and Min/Max are uniformly 0 and 100, which only reflect the theoretical scoring range of the EORTC QLQ‑C30. Replace these with more informative distributional statistics such as the median and interquartile range (IQR) and/or 95% confidence intervals for the mean. Given the large SDs and likely skewness or floor/ceiling effects for some scales, median and IQR would be especially informative and align with best practice for HRQoL data.

We thank the reviewer for this valuable suggestion. In response, Table 3 has been revised by removing the N, minimum, and maximum columns, as these reflected the uniform sample size and the theoretical scoring range of the EORTC QLQ-C30 scale and therefore did not provide meaningful distributional information. These have been replaced with median and interquartile range (IQR), in addition to mean ± standard deviation for completeness.

This revision improves the presentation of the data by better reflecting the observed distribution of health-related quality of life scores. Given the skewed distributions and the presence of floor and ceiling effects in several scales, the inclusion of median and IQR provides a more appropriate and robust summary of central tendency and dispersion, in line with recommended reporting practices for HRQoL data.

**Comment 8 — Analytical sample for regression (Table 5)**

The heading of Table 5 reads “Multiple Linear Regression Analysis of Factors Associated with the Number of Non‑oncological Medications (n = 132),” which suggests the model was run only among the 132 participants with excessive polypharmacy (>10). If so, this is a major problem: predictors of medication count should be modelled in the full sample (n = 234). Restricting the analysis to those already above a threshold introduces selection bias and limits generalisability. Please clarify which analytical sample was used; if the regression was restricted to n = 132, re‑run it on the complete sample and update the table and model statistics (F, R², degrees of freedom) so they are internally consistent.

Thank you for this important comment. We confirm that the multiple linear regression analysis was re-run using the full analytical sample (n = 234) rather than a restricted subgroup. The previous heading indicating *n = 132* was incorrect and has been revised accordingly to avoid misunderstanding.

The updated analysis includes all eligible participants and examines predictors of the total number of non-oncological medications in the complete cohort. The revised model is now internally consistent, with updated degrees of freedom, model statistics (F, R², Adjusted R²), and regression coefficients reflecting the full sample.

We have updated Table 5 and 6 and all related results in the manuscript to ensure methodological clarity and eliminate potential selection bias. The revised model demonstrates that the analysis is generalizable to the entire study population.
