# Supplementary material for: Polypharmacy and quality of life in Yemeni cancer patients undergoing chemotherapy: A cross-sectional study
Source: PLOS Glob Public Health. 2026 Sep 28;6(9):e0006281. doi: 10.1371/journal.pgph.0006281 (PMC13618909; doi:10.1371/journal.pgph.0006281)
Supplement: Supplementary file 5 [file pgph.0006281.s006.docx]

04-08-2026

Dear Dr. Catherine Kyobutungi

Editor-in-Chief
PLOS Global Public Health

Thank you for your decision letter dated 27 July 2026 and for giving us the opportunity to submit a revised version of our manuscript, “**Polypharmacy and Quality of Life in Yemeni Cancer Patients Undergoing Chemotherapy: A Cross-Sectional Study**”

We are sincerely grateful to your valuable comments. We carefully considered each point raised and have revised the manuscript accordingly. We believe these changes have further strengthened the clarity and rigor of the work. As the first study of its kind in Yemen, it offers valuable insights that will enrich the broader scientific understanding of this topic.

Enclosed is our detailed point-by-point response to the editor’s and reviewers’ comments. For ease of review, major revisions in the manuscript have been highlighted in Yellow.

We truly appreciate your positive decision and the opportunity to resubmit. We hope the revised manuscript will now meet the expectations for publication in PLOS Global Public Health.

We look forward to hearing from you.

Yours Sincerely,

Sayida Al-Jamei (PhD)

Department of Clinical Pharmacy. School of Medical Science and Pharmacy, Lebanese International University, Sana’a, Yemen

Email: [saydaahmed@yahoo.com](mailto:saydaahmed@yahoo.com)

The response:

Thank you so much for this decision. We provided below one by one responses to the reviewer’s comments (blue color)

**Additional Editor Comments:**

Although the manuscript has improved and several previous comments have been addressed, important inconsistencies remain in the exposure definitions, analytical methods and reporting of results. These concerns appear potentially correctable but require careful reanalysis and reconciliation across the abstract, Methods, tables and Discussion before the manuscript can be considered for publication. These are several issues that remain unresolved or are internally inconsistent.

Definition of polypharmacy

The definition has not yet been harmonised throughout the manuscript. The abstract defines polypharmacy as ≥5 non-oncological medications but reports the prevalence using >5. The Study Instruments section also uses “more than five,” whereas the Study Analysis section uses “five or more (≥5).” Excessive polypharmacy similarly alternates between >10 and ≥10. Please select the intended thresholds, apply them consistently throughout, and verify whether the reported prevalence estimates change.

Thank you for this valuable comment. We have carefully reviewed the manuscript and standardized the definition of polypharmacy throughout. Polypharmacy is now consistently defined as the concurrent use of five or more (≥5) non-oncological medications, and excessive polypharmacy as the concurrent use of ten or more (≥10) non-oncological medications, in accordance with the cited reference. We also verified all analyses and prevalence estimates, which have been updated where necessary to ensure consistency throughout the abstract, Methods, Results, and all other relevant sections.

Outcome used in the polypharmacy regression

The Study Analysis section describes the second regression model as evaluating predictors of “excessive polypharmacy,” defined as >10 medications. However, Table 5 presents a linear regression of the continuous number of non-oncological medications. Please clarify the actual dependent variable. If the outcome is medication count, this should be stated consistently; if it is the dichotomous excessive-polypharmacy variable, an appropriate binary regression model would be required.

Thank you for this important observation. We have revised the manuscript to clarify the dependent variable used in the second regression model. The outcome variable was the continuous number of non-oncological medications, and therefore a multiple linear regression analysis was appropriately performed. We have corrected the description in the Study Analysis section to ensure consistency with Table 5 and have removed the incorrect statement indicating that the model evaluated predictors of excessive polypharmacy. The terminology has been standardized throughout the manuscript to avoid any ambiguity.

Analytical sample and missing data

Table 5 now reports an analytical sample of 234 participants, but Table 1 shows missing data for age, education, residence and time since diagnosis—all variables included in the regression. Please explain how the full sample was retained, including whether missing values were imputed, recoded or handled in another way.

Thank you for highlighting this important issue. We have clarified the handling of missing data in the revised manuscript. Table 1 already reports the available observations for variables with missing values, including age (n = 229), education (n = 233), residence (n = 233), cancer stage (n = 151), and time since diagnosis (n = 228).

The variables included in the final multivariable regression models were age group, gender, marital status, occupation, education level, residence, monthly income, comorbidity status, duration since cancer diagnosis, and excessive polypharmacy status. Cancer stage was not included in the regression models because of substantial missing data (available for only 151 participants), which would have considerably reduced the analytical sample size and statistical power.

The Global Health Status/QoL regression model included 234 participants, as reported in Table 6. Missing values reported in Table 1 were limited and did not affect the analytical sample included in the regression model. No imputation method was applied.

Cancer stage was not included as an independent variable in the regression models because of substantial missing information (available for only 151 participants; 83 missing values). Including this variable would have considerably reduced the analytical sample size and statistical power; therefore, cancer stage was excluded from the multivariable analyses.

The Methods section has been revised to clarify the variables included in the regression analyses, the handling of missing data, and the rationale for excluding variables with substantial missing information.

Internal inconsistencies in the QoL model

Table 6 reports (F(11,222)), implying 11 predictors, but only 10 predictor coefficients are shown. Time since diagnosis is described in the text as included in the model but does not appear in the table. In addition, the standard error of the estimate is reported as 24.99 in the Results and 25.60 beneath Table 6. Please reconcile these figures and present all variables included in the final model.

Thank you for highlighting this inconsistency. We reviewed the QoL regression model and identified that duration since diagnosis was included in the original SPSS model but was inadvertently omitted from Table 6. The table has been corrected to include all variables entered into the final model, including duration since diagnosis (>1 year).

The Methods section has also been revised to include duration since diagnosis among the independent variables. The model statistics have been verified, and the reported F(11,222) value and standard error of the estimate (25.60) are now consistent with the final regression model.

Interpretation of comorbidity

The Discussion states that the absence of comorbidities was associated with fewer non-oncological medications and that this confirms multimorbidity as a major driver of polypharmacy. However, Table 5 shows that the comorbidity variable was not statistically significant ((B=-0.417,\ p=.416)). This interpretation should therefore be revised.

Thank you for highlighting this issue. We agree that the previous interpretation was not consistent with the regression findings. Although the absence of comorbidities showed a negative coefficient in the medication burden model (B = -0.417), this association was not statistically significant (p = 0.416). Therefore, the statement suggesting that absence of comorbidities was associated with fewer non-oncological medications and that our findings confirmed multimorbidity as a major driver of polypharmacy has been revised.

The Discussion section has been modified to clarify that, in this study, comorbidity status was not an independent factor associated with the number of non-oncological medications. The relationship between multimorbidity and polypharmacy described in previous literature is acknowledged, while our findings should be interpreted cautiously given the lack of statistical significance in the present analysis.

Abstract and Table 4

The abstract reports the 95% confidence interval for the mean QoL difference as −13.11 to 6.41, whereas Table 4 reports −10.58 to 3.87. Please confirm the correct interval and update all relevant sections, including the abstract entered in the submission system. The submission-system abstract also appears to retain regression estimates from an earlier analysis.

Thank you for identifying this inconsistency. We reviewed the analysis and confirmed that the correct 95% confidence interval for the mean difference in Global Health Status/QoL between patients with and without excessive polypharmacy is −10.58 to 3.87 (mean difference = −3.35; p = 0.361), as reported in Table 4.

The confidence interval reported in the abstract (−13.11 to 6.41) was from an earlier analysis and has been corrected. The Abstract section in the revised manuscript and the abstract submitted through the submission system have both been updated to reflect the final analysis results. We also reviewed the regression estimates in the submission-system abstract and updated them to ensure consistency with the final regression models presented in the revised manuscript.

Reporting of percentages

The response states that every percentage is now accompanied by its numerator and denominator, but this has not been consistently implemented. This is particularly important for variables with missing data. For example, the age percentages in the narrative differ from those in Table 1 because different denominators appear to have been used. Please report (n/N) alongside percentages and clearly distinguish percentages based on the full sample from valid percentages based on non-missing observations.

Thank you for highlighting this issue. We have revised the reporting of percentages throughout the manuscript to ensure that the corresponding denominators are clear. In the Results section, percentages for variables with missing data are now reported based on the available observations (e.g., age: 109/229; 47.6%). Table 1 already included footnotes indicating the number of available observations for variables with missing values, and these have been retained. The narrative descriptions have been revised to ensure consistency with the denominators reported in Table 1.

Clinical variables

Adding cancer stage to Table 1 is helpful, and the explanation for excluding it from the regression is reasonable given the amount of missing data. However, the requested detailed categories of comorbidities have not been provided. Please add these categories if available or state clearly that sufficiently detailed information was unavailable. The limitations should also consistently acknowledge the absence of treatment intensity and other clinically important confounders.

Thank you for this helpful suggestion. We have revised Table 1 to include the available categories of comorbidities. Specifically, hypertension, diabetes mellitus, heart disease, kidney disease, and other reported comorbid conditions are now presented to provide a more detailed description of the study population.

We have also revised the Limitations section to acknowledge that treatment intensity and other clinically important factors that may influence medication burden and quality of life were not available in our dataset and therefore could not be included in the multivariable analyses. We recognize that the absence of these variables may have resulted in residual confounding and have highlighted this as a limitation of the study.

Sample-size justification

The revised section reports a post-hoc power assessment but does not provide the exact (f^2), exact estimated power or number of predictors for each model. The stated Green rule is also now (50+10m), whereas the reviewer referred to (50+8m), without an explanation for the change. Please provide the calculation transparently or limit the section to an honest acknowledgement that no a priori power calculation was performed.

Thank you for highlighting this issue. We agree that the previous description of the post-hoc power assessment was insufficiently detailed and did not address the reviewer’s request for a formal a priori power calculation. Therefore, the post-hoc power assessment has been removed from the revised manuscript.

We have clarified the sample-size justification by reporting that Green’s rule of thumb for multiple regression (N ≥ 50 + 8m) was used as a sample-size adequacy assessment, where m represents the number of predictors included in each regression model. For the Global Health Status/QoL model, 11 predictors were included, resulting in a minimum required sample size of 138 participants. For the medication burden model, 10 predictors were included, resulting in a minimum required sample size of 130 participants. The final analytical sample of 234 participants exceeded these recommended thresholds.

The previous manuscript incorrectly reported Green’s rule as N ≥ 50 + 10m due to a typographical error. This has been corrected to N ≥ 50 + 8m according to Green’s original recommendation and the cited reference. We have also added a statement in the Limitations section acknowledging that a formal a priori power calculation based on an expected effect size, alpha level, and desired power was not performed before data collection.

Journal requirements

The Ethics Statement was requested at the beginning of the Methods under the subheading “Ethics Statement.” It currently appears as Section 2.3 under a different heading. The manuscript’s Data Availability Statement refers to the Supporting Information, but the submission-system statement still says that the data are available “within the article.” These should be aligned with the journal’s instructions.

Thank you for highlighting these points. We have revised the manuscript to ensure compliance with the journal requirements. The Ethics Statement has been moved to the beginning of the Methods section and is now presented as a separate subsection (Section 2.1: Ethics Statement).

In addition, the Data Availability Statement has been revised to align with the submission-system statement and now consistently indicates that the data supporting the findings are available in the manuscript and its Supporting Information files. The corresponding statement entered in the submission system has also been updated accordingly.
