# Supplementary material for: Polypharmacy and quality of life in Yemeni cancer patients undergoing chemotherapy: A cross-sectional study
Source: PLOS Glob Public Health. 2026 Sep 28;6(9):e0006281. doi: 10.1371/journal.pgph.0006281 (PMC13618909; doi:10.1371/journal.pgph.0006281)
Supplement: Supplementary file 6 [file pgph.0006281.s007.docx]

28-08-2026

Dear Dr. Catherine Kyobutungi

Editor-in-Chief
PLOS Global Public Health

Thank you for your decision letter dated 18 August 2026 and for giving us the opportunity to submit a revised version of our manuscript, “**Polypharmacy and Quality of Life in Yemeni Cancer Patients Undergoing Chemotherapy: A Cross-Sectional Study**”

We are sincerely grateful to your valuable comments. We carefully considered each point raised and have revised the manuscript accordingly. We believe these changes have further strengthened the clarity and rigor of the work. As the first study of its kind in Yemen, it offers valuable insights that will enrich the broader scientific understanding of this topic.

Enclosed is our detailed point-by-point response to the editor’s comments. For ease of review, major revisions in the manuscript have been highlighted in Yellow.

We truly appreciate your positive decision and the opportunity to resubmit. We hope the revised manuscript will now meet the expectations for publication in PLOS Global Public Health.

We look forward to hearing from you.

Yours Sincerely,

Sayida Al-Jamei (PhD)

Department of Clinical Pharmacy. School of Medical Science and Pharmacy, Lebanese International University, Sana’a, Yemen

Email: [saydaahmed@yahoo.com](mailto:saydaahmed@yahoo.com)

The response:

Thank you so much for this decision. We provided below one by one responses to the reviewer’s comments (blue color)

**Additional Editor Comments:**

**Additional Editor Comments (if provided):**

The manuscript has improved substantially, and all previous revisions are accepted except the following three points:

Polypharmacy thresholds

The definitions remain inconsistent. See the submission-system abstract and manuscript lines 31–32, 157–158 (lime 158 should be 10 or more than...) and 191–193. In addition, lines 283–289 define excessive polypharmacy as ≥10 medications but describe the comparison group as ≤10; this should be <10.

Thank you for identifying this remaining inconsistency. We have carefully reviewed and corrected the definitions of polypharmacy and excessive polypharmacy throughout the manuscript and submission-system abstract.

We have standardized the definitions as follows: polypharmacy was defined as the use of ≥5 medications, while excessive polypharmacy was defined as the use of ≥10 medications. Accordingly, the comparison group for excessive polypharmacy has been corrected from ≤10 medications to <10 medications.

The relevant statements in the submission-system abstract and manuscript Lines 31–32, 157–158, 191–193, and 283–289 have been revised accordingly. In particular, Line 158 has been corrected to “10 or more medications (≥10)”, and the comparison group in Lines 283–289 is now correctly stated as <10 medications.

We sincerely thank the Editor for pointing out this inconsistency. The manuscript has been carefully checked to ensure that the polypharmacy thresholds are now consistent throughout the abstract, Methods, Results, and other relevant sections.

Regression sample

Lines 199–203 state that complete cases were used, while Table 1 reports missing predictor data (lines 222–226). However, Tables 5 and 6 still report (n=234) (lines 312–314 and 339–341). Please reconcile the analytical sample sizes and clarify how the missing values were handled.

Thank you for highlighting this apparent inconsistency. We have carefully reviewed the analytical sample sizes and clarified the handling of missing data in the regression analyses.

We have revised Lines 199–203 to remove the statement that “available complete cases” were used, as this wording could incorrectly imply that participants with any missing predictor data were excluded. Cancer stage was excluded from the multivariable regression analyses because data were available for only 151 participants (83 missing values), which would have substantially reduced the analytical sample size and statistical power. The remaining predictor variables had minimal missing data, and the final regression models included all 234 participants. Accordingly, the sample sizes reported in Tables 5 and 6 (n = 234) are consistent with the actual analytical sample and the reported model statistics.

We have also clarified in the Limitations section that information on treatment intensity and other clinically relevant factors, including performance status and detailed treatment-related characteristics, was not available and therefore could not be included in the analyses. Cancer stage was likewise not included because of incomplete clinical documentation and substantial missing data. These unmeasured factors may have influenced medication burden and quality of life and represent potential sources of residual confounding.

We appreciate the Reviewer’s careful observation, which helped us improve the clarity and transparency of the missing-data handling and regression analyses.

Remaining reporting inconsistencies

Line 329 states age ≥50 years, whereas Table 6 uses ≥51 years. Please also complete the requested (n/N) reporting for narrative percentages, particularly in lines 234–250 and 261–264.

Thank you for identifying these remaining reporting inconsistencies.

First, the statement in Line 329 reporting age ≥50 years was a typographical error. It has been corrected to age ≥51 years, consistent with the categorization presented in Table 6.

Second, we have revised the narrative reporting of percentages to provide the corresponding numerator and denominator (n/N), particularly in Lines 234–250 and 261–264, as requested. We have also reviewed the surrounding Results text and standardized the reporting format where appropriate. The denominators were specified according to the number of participants with available data when applicable (e.g., cancer stage, n/N = 130/151 and 21/151; disease duration, n/N = 162/228), while variables with complete data were reported using N = 234.

We appreciate the Editor’s careful review, which has helped us improve the accuracy, transparency, and consistency of the Results section.
