# Supplementary material for: Polypharmacy and quality of life in Yemeni cancer patients undergoing chemotherapy: A cross-sectional study
Source: PLOS Glob Public Health. 2026 Sep 28;6(9):e0006281. doi: 10.1371/journal.pgph.0006281 (PMC13618909; doi:10.1371/journal.pgph.0006281)
Supplement: Peer review history

# Peer review: Decision Letter 0

**DOI:** 10.1371/journal.pgph.0006281.r001

**Authors:** Kelechi Elizabeth Oladimeji

20 May 2026

PGPH-D-26-01135

Polypharmacy and Quality of Life in Yemeni Cancer Patients Undergoing Chemotherapy: A Cross-Sectional Study

PLOS Global Public Health

Dear Dr. Al-Jamei,

Thank you for submitting your manuscript to PLOS Global Public Health. After careful consideration, we feel that it has merit but does not fully meet PLOS Global Public Health’s publication criteria as it currently stands. Therefore, we invite you to submit a revised version of the manuscript that addresses the points raised during the review process.

EDITOR: The reviewers have provided helpful feedback that should be carefully considered in revising the manuscript. A further decision on the review outcome will require a clear point-by-point response to the methodological, statistical, reporting, and presentation issues raised, with corresponding revisions to ensure that the conclusions are fully supported by the data and that the manuscript meets PLOS Global Public Health’s publication criteria.

Please submit your revised manuscript by Jul 19 2026 11:59PM. If you will need more time than this to complete your revisions, please reply to this message or contact the journal office at globalpubhealth@plos.org. When you're ready to submit your revision, log on to https://www.editorialmanager.com/pgph/ and select the 'Submissions Needing Revision' folder to locate your manuscript file.

Please include the following items when submitting your revised manuscript:

- A letter that responds to each point raised by the editor and reviewer(s). You should upload this letter as a separate file labeled 'Response to Reviewers'.
- A marked-up copy of your manuscript that highlights changes made to the original version. You should upload this as a separate file labeled 'Revised Manuscript with Track Changes'.
- An unmarked version of your revised paper without tracked changes. You should upload this as a separate file labeled 'Manuscript'.

Guidelines for resubmitting your figure files are available below the reviewer comments at the end of this letter.

As the corresponding author, your ORCID iD is verified in the submission system and will appear in the published article. PLOS supports the use of ORCID, and we encourage all coauthors to register for an ORCID iD and use it as well. Please encourage your coauthors to verify their ORCID iD within the submission system before final acceptance, as unverified ORCID iDs will not appear in the published article. *Only*  the individual author can complete the verification step; PLOS staff *cannot*  verify ORCID iDs on behalf of authors.

We look forward to receiving your revised manuscript.

Kind regards,

Kelechi Elizabeth Oladimeji

Guest Editor

PLOS Global Public Health

Journal Requirements:

1. Please ensure that your Ethics Statement is available in its entirety at the beginning of your Methods section, under a subheading 'Ethics Statement'.

2. Tables should not be uploaded as individual files. Please remove these files and include the Tables in your manuscript file as editable, cell-based objects. For more information about how to format tables, see our guidelines: [LINK]

https://journals.plos.org/globalpublichealth/s/tables

3. We note that your Data Availability Statement is currently as follows: "The authors confirm that the data supporting the findings of this study are available within the article"

Please confirm at this time whether or not your submission contains all raw data required to replicate the results of your study. Authors must share the “minimal data set” for their submission. PLOS defines the minimal data set to consist of the data required to replicate all study findings reported in the article, as well as related metadata and methods (https://journals.plos.org/plosone/s/data-availability#loc-minimal-data-set-definition).

For example, authors should submit the following data:

- The values behind the means, standard deviations and other measures reported;

- The values used to build graphs;

- The points extracted from images for analysis.

Authors do not need to submit their entire data set if only a portion of the data was used in the reported study.

If your submission does not contain these data, please either upload them as Supporting Information files or deposit them to a stable, public repository and provide us with the relevant URLs, DOIs, or accession numbers. For a list of recommended repositories, please see https://journals.plos.org/plosone/s/recommended-repositories.

If the reviewer comments include a recommendation to cite specific previously published works, please review and evaluate these publications to determine whether they are relevant and should be cited. There is no requirement to cite these works unless the editor has indicated otherwise.

Additional Editor Comments (if provided):

[Note: HTML markup is below. Please do not edit.]

Reviewers' comments:

Reviewer's Responses to Questions

**Comments to the Author**

1. Does this manuscript meet PLOS Global Public Health’s publication criteria?

Reviewer #1: Partly

Reviewer #2: Yes

Reviewer #3: Yes

Reviewer #4: Partly

**********

2. Has the statistical analysis been performed appropriately and rigorously?

Reviewer #1: No

Reviewer #2: Yes

Reviewer #3: Yes

Reviewer #4: No

**********

3. Have the authors made all data underlying the findings in their manuscript fully available (please refer to the Data Availability Statement at the start of the manuscript PDF file)?

The PLOS Data policy

Reviewer #1: No

Reviewer #2: Yes

Reviewer #3: Yes

Reviewer #4: Yes

**********

4. Is the manuscript presented in an intelligible fashion and written in standard English?

Reviewer #1: Yes

Reviewer #2: Yes

Reviewer #3: Yes

Reviewer #4: No

**********

Reviewer #1: Thank you for the opportunity to review this manuscript. The study addresses an important and underexplored topic, particularly within a resource-limited and conflict-affected setting such as Yemen, and contributes valuable preliminary data on polypharmacy and quality of life among cancer patients undergoing chemotherapy.

Overall, the manuscript is well structured and clearly written, with a logical flow from introduction to conclusion. However, several areas require further clarification and strengthening. The interpretation of the findings, particularly the lack of association between polypharmacy and quality of life, should be presented more cautiously given the limited variability in exposure, as the vast majority of participants were already experiencing polypharmacy. This may have limited the ability to detect meaningful differences. In addition, further justification is needed for the definition of polypharmacy focusing exclusively on non-oncological medications, as this differs from commonly used definitions in the literature.

There are also important considerations related to potential confounding that should be more explicitly acknowledged. Key clinical variables such as cancer stage, treatment intensity, and symptom severity were not included in the analysis, although they are likely to have a significant impact on quality of life outcomes. The relatively low explanatory power of the regression models further suggests that additional relevant factors may not have been captured.

The manuscript would also benefit from a more in-depth discussion of contextual factors, particularly the role of gender. The finding that female patients experience lower quality of life and higher levels of polypharmacy should be interpreted within the broader sociocultural and structural context of Yemen, where gender-related disparities in healthcare access, financial autonomy, and social support may act as important confounders.

In addition, ethical considerations could be more comprehensively addressed. While ethical approval and consent procedures are mentioned, further reflection on participant vulnerability, the clinical setting of recruitment, and measures taken to ensure voluntary participation would strengthen the manuscript.

Finally, some minor revisions related to clarity, consistency in terminology, and table presentation are recommended to improve readability.

Please see the attached document for more detailed comments and specific suggestions for revision.

Reviewer #2: This is an original recent work. No searched articles are similar or replicates of this study. The study was carefully planned, executed and recorded. The conclusions align well with the findings and the objectives of the study. Appropriate ethical procedures were taken and reported. The whole presentation is logical and intelligible. Data availability to the public has been declared.

Reviewer #3: Thank you for inviting me to review this manuscript which makes a meaningful contribution to the literature on the prevalence and determinants of polypharmacy among cancer patients undergoing chemotherapy in Yemen. The analysis and findings are valuable; however, several methodological clarifications and textual corrections are needed to improve clarity, consistency, and presentation. I hope that my comments below would assist the authors in revising and further strengthening the manuscript.

Reviewer #4: It is a relevant piece of work. The findings shall be of use to the prescribers and very likely improve the safer use of medications.

It is proposed to address the comments in order to be scientifically sound.

**********

what does this mean?). If published, this will include your full peer review and any attached files.

**Do you want your identity to be public for this peer review?**  If you choose “no”, your identity will remain anonymous but your review may still be made public.

For information about this choice, including consent withdrawal, please see our Privacy Policy

Reviewer #1: No

Reviewer #2: **Yes:**  Sam Agatre Okuonzi

(This my full name)

Reviewer #3: No

Reviewer #4: No

**********

[NOTE: If reviewer comments were submitted as an attachment file, they will be attached to this email and accessible via the submission site. Please log into your account, locate the manuscript record, and check for the action link "View Attachments". If this link does not appear, there are no attachment files.]

Attachment Submitted filename: Reviewer Comments_PGPH-D-26-01135_reviewer.docx

Attachment Submitted filename: Report..pdf

Attachment Submitted filename: Comments PGPH.docx

# Author comment: Author response to Decision Letter 1

**DOI:** 10.1371/journal.pgph.0006281.r002

28 Jun 2026

Attachment Submitted filename: The response.docx

# Editor report: Decision Letter 1

**DOI:** 10.1371/journal.pgph.0006281.r003

**Authors:** Kelechi Elizabeth Oladimeji

27 Jul 2026

PGPH-D-26-01135R1

Polypharmacy and Quality of Life in Yemeni Cancer Patients Undergoing Chemotherapy: A Cross-Sectional Study

PLOS Global Public Health

Dear Dr. Al-Jamei,

Thank you for submitting your manuscript to PLOS Global Public Health. After careful consideration, we feel that it has merit but does not fully meet PLOS Global Public Health’s publication criteria as it currently stands. Therefore, we invite you to submit a revised version of the manuscript that addresses the points raised during the review process.

Please submit your revised manuscript by Sep 25 2026 11:59PM. If you will need more time than this to complete your revisions, please reply to this message or contact the journal office at globalpubhealth@plos.org. When you're ready to submit your revision, log on to https://www.editorialmanager.com/pgph/ and select the 'Submissions Needing Revision' folder to locate your manuscript file.

Please include the following items when submitting your revised manuscript:

- A letter that responds to each point raised by the editor and reviewer(s). You should upload this letter as a separate file labeled 'Response to Reviewers'.
- A marked-up copy of your manuscript that highlights changes made to the original version. You should upload this as a separate file labeled 'Revised Manuscript with Track Changes'.
- An unmarked version of your revised paper without tracked changes. You should upload this as a separate file labeled 'Manuscript'.

Guidelines for resubmitting your figure files are available below the reviewer comments at the end of this letter.

As the corresponding author, your ORCID iD is verified in the submission system and will appear in the published article. PLOS supports the use of ORCID, and we encourage all coauthors to register for an ORCID iD and use it as well. Please encourage your coauthors to verify their ORCID iD within the submission system before final acceptance, as unverified ORCID iDs will not appear in the published article. *Only*  the individual author can complete the verification step; PLOS staff *cannot*  verify ORCID iDs on behalf of authors.

We look forward to receiving your revised manuscript.

Kind regards,

Kelechi Elizabeth Oladimeji

Guest Editor

PLOS Global Public Health

**Journal Requirements:**

If the reviewer comments include a recommendation to cite specific previously published works, please review and evaluate these publications to determine whether they are relevant and should be cited. There is no requirement to cite these works unless the editor has indicated otherwise.

**Additional Editor Comments (if provided):**

Although the manuscript has improved and several previous comments have been addressed, important inconsistencies remain in the exposure definitions, analytical methods and reporting of results. These concerns appear potentially correctable but require careful reanalysis and reconciliation across the abstract, Methods, tables and Discussion before the manuscript can be considered for publication. These are several issues that remain unresolved or are internally inconsistent.

Definition of polypharmacy

The definition has not yet been harmonised throughout the manuscript. The abstract defines polypharmacy as ≥5 non-oncological medications but reports the prevalence using >5. The Study Instruments section also uses “more than five,” whereas the Study Analysis section uses “five or more (≥5).” Excessive polypharmacy similarly alternates between >10 and ≥10. Please select the intended thresholds, apply them consistently throughout, and verify whether the reported prevalence estimates change.

Outcome used in the polypharmacy regression

The Study Analysis section describes the second regression model as evaluating predictors of “excessive polypharmacy,” defined as >10 medications. However, Table 5 presents a linear regression of the continuous number of non-oncological medications. Please clarify the actual dependent variable. If the outcome is medication count, this should be stated consistently; if it is the dichotomous excessive-polypharmacy variable, an appropriate binary regression model would be required.

Analytical sample and missing data

Table 5 now reports an analytical sample of 234 participants, but Table 1 shows missing data for age, education, residence and time since diagnosis—all variables included in the regression. Please explain how the full sample was retained, including whether missing values were imputed, recoded or handled in another way.

Internal inconsistencies in the QoL model

Table 6 reports (F(11,222)), implying 11 predictors, but only 10 predictor coefficients are shown. Time since diagnosis is described in the text as included in the model but does not appear in the table. In addition, the standard error of the estimate is reported as 24.99 in the Results and 25.60 beneath Table 6. Please reconcile these figures and present all variables included in the final model.

Interpretation of comorbidity

The Discussion states that the absence of comorbidities was associated with fewer non-oncological medications and that this confirms multimorbidity as a major driver of polypharmacy. However, Table 5 shows that the comorbidity variable was not statistically significant ((B=-0.417,\ p=.416)). This interpretation should therefore be revised.

Abstract and Table 4

The abstract reports the 95% confidence interval for the mean QoL difference as −13.11 to 6.41, whereas Table 4 reports −10.58 to 3.87. Please confirm the correct interval and update all relevant sections, including the abstract entered in the submission system. The submission-system abstract also appears to retain regression estimates from an earlier analysis.

Reporting of percentages

The response states that every percentage is now accompanied by its numerator and denominator, but this has not been consistently implemented. This is particularly important for variables with missing data. For example, the age percentages in the narrative differ from those in Table 1 because different denominators appear to have been used. Please report (n/N) alongside percentages and clearly distinguish percentages based on the full sample from valid percentages based on non-missing observations.

Clinical variables

Adding cancer stage to Table 1 is helpful, and the explanation for excluding it from the regression is reasonable given the amount of missing data. However, the requested detailed categories of comorbidities have not been provided. Please add these categories if available or state clearly that sufficiently detailed information was unavailable. The limitations should also consistently acknowledge the absence of treatment intensity and other clinically important confounders.

Sample-size justification

The revised section reports a post-hoc power assessment but does not provide the exact (f^2), exact estimated power or number of predictors for each model. The stated Green rule is also now (50+10m), whereas the reviewer referred to (50+8m), without an explanation for the change. Please provide the calculation transparently or limit the section to an honest acknowledgement that no a priori power calculation was performed.

Journal requirements

The Ethics Statement was requested at the beginning of the Methods under the subheading “Ethics Statement.” It currently appears as Section 2.3 under a different heading. The manuscript’s Data Availability Statement refers to the Supporting Information, but the submission-system statement still says that the data are available “within the article.” These should be aligned with the journal’s instructions.

[Note: HTML markup is below. Please do not edit.]

**Reviewers' comments:**

[NOTE: If reviewer comments were submitted as an attachment file, they will be attached to this email and accessible via the submission site. Please log into your account, locate the manuscript record, and check for the action link "View Attachments". If this link does not appear, there are no attachment files.]

**Figure Resubmissions:**

# Author comment: Author response to Decision Letter 2

**DOI:** 10.1371/journal.pgph.0006281.r004

4 Aug 2026

Attachment Submitted filename: The response 2.docx

# Editor report: Decision Letter 2

**DOI:** 10.1371/journal.pgph.0006281.r005

**Authors:** Kelechi Elizabeth Oladimeji

17 Aug 2026

PGPH-D-26-01135R2

Polypharmacy and Quality of Life in Yemeni Cancer Patients Undergoing Chemotherapy: A Cross-Sectional Study

PLOS Global Public Health

Dear Dr. Al-Jamei,

Thank you for submitting your manuscript to PLOS Global Public Health. After careful consideration, we feel that it has merit but does not fully meet PLOS Global Public Health’s publication criteria as it currently stands. Therefore, we invite you to submit a revised version of the manuscript that addresses the points raised during the review process.

==============================

EDITOR: Please see additional editorial comments below.

==============================

Please submit your revised manuscript by Sep 16 2026 11:59PM. If you will need more time than this to complete your revisions, please reply to this message or contact the journal office at globalpubhealth@plos.org. When you're ready to submit your revision, log on to https://www.editorialmanager.com/pgph/ and select the 'Submissions Needing Revision' folder to locate your manuscript file.

Please include the following items when submitting your revised manuscript:

- A letter that responds to each point raised by the editor and reviewer(s). You should upload this letter as a separate file labeled 'Response to Reviewers'.
- A marked-up copy of your manuscript that highlights changes made to the original version. You should upload this as a separate file labeled 'Revised Manuscript with Track Changes'.
- An unmarked version of your revised paper without tracked changes. You should upload this as a separate file labeled 'Manuscript'.

Guidelines for resubmitting your figure files are available below the reviewer comments at the end of this letter.

As the corresponding author, your ORCID iD is verified in the submission system and will appear in the published article. PLOS supports the use of ORCID, and we encourage all coauthors to register for an ORCID iD and use it as well. Please encourage your coauthors to verify their ORCID iD within the submission system before final acceptance, as unverified ORCID iDs will not appear in the published article. *Only*  the individual author can complete the verification step; PLOS staff *cannot*  verify ORCID iDs on behalf of authors.

We look forward to receiving your revised manuscript.

Kind regards,

Kelechi Elizabeth Oladimeji

Guest Editor

PLOS Global Public Health

**Journal Requirements:**

If the reviewer comments include a recommendation to cite specific previously published works, please review and evaluate these publications to determine whether they are relevant and should be cited. There is no requirement to cite these works unless the editor has indicated otherwise.

Please review your reference list to ensure that it is complete and correct. If you have cited papers that have been retracted, please include the rationale for doing so in the manuscript text, or remove these references and replace them with relevant current references. Any changes to the reference list should be mentioned in the rebuttal letter that accompanies your revised manuscript. If you need to cite a retracted article, indicate the article’s retracted status in the References list and also include a citation and full reference for the retraction notice.

**Additional Editor Comments (if provided):**

The manuscript has improved substantially, and all previous revisions are accepted except the following three points:

Polypharmacy thresholds

The definitions remain inconsistent. See the submission-system abstract and manuscript lines 31–32, 157–158 (lime 158 should be 10 or more than...) and 191–193. In addition, lines 283–289 define excessive polypharmacy as ≥10 medications but describe the comparison group as ≤10; this should be <10.

Regression sample

Lines 199–203 state that complete cases were used, while Table 1 reports missing predictor data (lines 222–226). However, Tables 5 and 6 still report (n=234) (lines 312–314 and 339–341). Please reconcile the analytical sample sizes and clarify how the missing values were handled.

Remaining reporting inconsistencies

Line 329 states age ≥50 years, whereas Table 6 uses ≥51 years. Please also complete the requested (n/N) reporting for narrative percentages, particularly in lines 234–250 and 261–264.

[Note: HTML markup is below. Please do not edit.]

**Reviewers' comments:**

[NOTE: If reviewer comments were submitted as an attachment file, they will be attached to this email and accessible via the submission site. Please log into your account, locate the manuscript record, and check for the action link "View Attachments". If this link does not appear, there are no attachment files.]

**Figure Resubmissions:**

# Author comment: Author response to Decision Letter 3

**DOI:** 10.1371/journal.pgph.0006281.r006

28 Aug 2026

Attachment Submitted filename: The response 3.docx

# Editor report: Decision Letter 3

**DOI:** 10.1371/journal.pgph.0006281.r007

**Authors:** Kelechi Elizabeth Oladimeji

7 Sep 2026

Polypharmacy and Quality of Life in Yemeni Cancer Patients Undergoing Chemotherapy: A Cross-Sectional Study

PGPH-D-26-01135R3

Dear Dr. Al-Jamei,

We are pleased to inform you that your manuscript 'Polypharmacy and Quality of Life in Yemeni Cancer Patients Undergoing Chemotherapy: A Cross-Sectional Study' has been provisionally accepted for publication in PLOS Global Public Health.

Before your manuscript can be formally accepted you will need to complete some formatting changes, which you will receive in a follow up email. A member of our team will be in touch with a set of requests.

Please note that your manuscript will not be scheduled for publication until you have made the required changes, so a swift response is appreciated.

IMPORTANT: The editorial review process is now complete. PLOS will only permit corrections to spelling, formatting or significant scientific errors from this point onwards. Requests for major changes, or any which affect the scientific understanding of your work, will cause delays to the publication date of your manuscript.

If your institution or institutions have a press office, please notify them about your upcoming paper to help maximize its impact. If they'll be preparing press materials, please inform our press team as soon as possible -- no later than 48 hours after receiving the formal acceptance. Your manuscript will remain under strict press embargo until 2 pm Eastern Time on the date of publication. For more information, please contact globalpubhealth@plos.org.

Thank you again for supporting Open Access publishing; we are looking forward to publishing your work in PLOS Global Public Health.

Best regards,

Kelechi Elizabeth Oladimeji

Guest Editor

PLOS Global Public Health

***********************************************************

Reviewer Comments (if any, and for reference):
